# Dynamics of Adaptation in Spatially Heterogeneous Metapopulations

**DOI:** 10.1371/journal.pone.0054697

**Published:** 2013-02-12

**Authors:** Julien Papaïx, Olivier David, Christian Lannou, Hervé Monod

**Affiliations:** 1 BIOlogie et GEstion des Risques en agriculture group (UR BIOGER-CPP), Plant Health and Environment division, Institut National de la Recherche Agronomique, Thiverval-Grignon, France; 2 Mathématiques et Informatique Appliquées group (UR MIAJ), Applied Mathematics and Informatics division, Institut National de la Recherche Agronomique, Jouy-en-Josas, France; University of California Santa Barbara, United States of America

## Abstract

The selection pressure experienced by organisms often varies across the species range. It is hence crucial to characterise the link between environmental spatial heterogeneity and the adaptive dynamics of species or populations. We address this issue by studying the phenotypic evolution of a spatial metapopulation using an adaptive dynamics approach. The singular strategy is found to be the mean of the optimal phenotypes in each habitat with larger weights for habitats present in large and well connected patches. The presence of spatial clusters of habitats in the metapopulation is found to facilitate specialisation and to increase both the level of adaptation and the evolutionary speed of the population when dispersal is limited. By showing that spatial structures are crucial in determining the specialisation level and the evolutionary speed of a population, our results give insight into the influence of spatial heterogeneity on the niche breadth of species.

## Introduction

Long term evolution of populations can lead to local adaptations to environmental conditions: organisms tend to have a higher fitness in their local habitat than organisms originating from other habitats. In natural populations, local adaptation is one of the main forces shaping biodiversity [1]–[3]. In agrosystems, man-driven selection leads to genotypes with high performances in given environments [4], [5] but also to pathogens that are adapted to their hosts and that are likely to be more damaging [6]. Understanding the mechanisms involved in adaptation is therefore an important challenge of evolutionary biology with potential application in agronomy.

Spatial heterogeneity of natural or agricultural systems results from externally imposed variations of environmental conditions (*e.g*. resources, physical characteristics). Divergent selection in space favours local adaptation to such spatial variations and the emergence of specialised organisms which is often pointed out as determinant for the maintenance of diversity since it allows the partitioning of resource use [7]. In a heterogeneous environment, however, migration from other habitats counter-balances local adaptation leading to a decrease in the mean fitness of local populations [8]. Migration between habitats at the global scale depends on the proportion of the different habitats, their spatial aggregation and the dispersal ability of the organism under investigation [9]. In natural ecosystems, habitat loss and fragmentation have strong effects on biodiversity and more particularly on the maintenance of specialist species [10]. Indeed, such species tolerate a narrower range of resources than generalist ones and are thus more susceptible to resource availability in the environment. Such a change in community structure along a gradient of landscape fragmentation is described by Devictor *et al*. [11] on birds. Based on a large scale bird survey, they studied the distribution of species in landscapes with varying levels of fragmentation and disturbances [12]. They found that specialist species tended to be located in less fragmented and less disturbed landscapes than generalists. More generally, the specialist decline due to anthropogenic disturbances is reported in various taxonomic groups and is known as biotic homogenisation of communities (see [13], for a review). In agrosystems, the shift from complex and diversified natural environments to much more simplified and genetically uniform agrosystems over vast areas [14] has facilitated the occurrence and spread of highly specialised and damaging plant pathogens [15]. The development of control strategies that hamper the evolution towards more damaging pathogens is a major challenge in crop protection.

Evolutionary responses to environmental disturbances are observed not only at the interspecific level but also at the intraspecific and intrapopulational ones [16]. For example, Barnagaud *et al*. [17] studied the variations of specialisation in response to habitat reduction for 94 bird species in France. They established that habitat specialisation decreased in 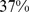 of bird species. In addition, this decrease in habitat specialisation was the most important for the most specialised species. In plant epidemiology, Papaïx *et al*. [18] have characterised the level of adaptation, at the scale of France, of the *Puccinia triticina* population (a fungus responsible of the wheat leaf rust) to several wheat varieties. They found that the rust population was composed of several genotypes with different degrees of specialisation (see also [19]). They also found that the amount of disease was influenced by variations in the frequencies of *P. triticina* specialist genotypes in the rust population, these variations being explained by modifications of the wheat landscape.

It is hence crucial to better characterise the link between spatial heterogeneity of the environment and the adaptive dynamics of species and populations. We address this question from a theoretical point of view and study how the components of spatial heterogeneity interplay to make a population evolve toward generalist or specialist phenotypes. We provide a general framework based on an adaptive dynamics approach [20]–[23] focused on the spatial description of the environment. Adaptive dynamics is a theoretical approach that refers to a set of techniques for studying long-term phenotypic changes of an evolving population. It requires two main assumptions: mutations are rare and they have small effects (evolution is gradual). The aim is then to compute the evolutionary equilibria (singular strategies) and to characterise their convergence stability (does a gradual evolution lead to them?) and evolutionary stability (are they resistant to invasion by any other phenotype?).

Several studies have investigated the effect of spatial heterogeneity on the evolution of specialisation using adaptive dynamics. In these studies, space has either been assumed to be a continuous domain or a discontinuous set of patches. For example, Doebeli & Dieckmann [24] considered a square area where environmental conditions changed gradually in one dimension. This assumption is suitable for modelling environmental heterogeneity due to changes in altitude, temperature, etc. Débarre & Gandon [25] considered a one-dimensional continuous space with two habitats that alternated. Geritz *et al*. [21], Meszéna *et al*. [26], Parvinen & Egas [27] and Ravigné *et al*. [28] considered metapopulation structures, consisting of a network of local populations interconnected by dispersal. In addition, space has either been assumed to be implicit, when the probability of migrating from one point to another does not depend on the distance between these points, or explicit. For example, Geritz *et al*. [21] considered space as implicit and used an uniform dispersal while Débarre & Gandon [25] considered a true spatial structure and addressed the problem by means of diffusion theory.

All these studies on the effect of spatial heterogeneity on the evolution of specialisation show that habitat differentiation and balanced habitat proportions favour the evolution of specialism while dispersal favours the evolution of generalism. They also show that the phenotype of the generalist is the mean of the optimal phenotypes in each habitat weighted by habitat proportions. However these results are incomplete for several reasons. First, they do not cover all possible situations. For example the phenotype of the generalist has not been defined when some patches receive fewer migrants than the other patches, which is a common feature of agricultural and fragmented landscapes. Indeed, spatial heterogeneity results in more or less connected habitat fragments which do not contribute to the same extend to the global network [29]. Second, the effect of the spatial distribution of habitats on the evolution of specialisation has been little studied. An exception is the study of Débarre & Gandon [25] which shows in a one-dimensional environment that evolution towards generalism is favored when habitats alternate frequently. Third, these studies mainly investigate whether evolution leads to specialists or to generalists, but when evolution leads to specialists, the phenotype of these specialists has been little studied. Exceptions are the studies of Geritz *et al*. [21] and Meszéna *et al*. [26] which show that specialist phenotypes are closer to the optimal phenotypes in each habitat when dispersal is limited and when habitats are differentiated. Fourth, the effect of spatial heterogeneity on the speed of adaptation has not been studied.

The stable coexistence of genotypes in spatially structured populations is also an important question in population genetics [30], [31]. In this approach, evolution is studied over shorter periods than in adaptive dynamics and a protected polymorphism occurs if selection is heterogeneous in space and is sufficiently strong relative to migration. When migration dominates selection, a rapid reduction of the gene-frequency differences among demes is expected. This panmictic evolution results in a unique mean fitness for the entire population. Eco-evolutionary models are also used to explore the dynamics of adaptation. Recently, Hanski *et al*. [32] proposed an eco-evolutionary dynamics model for a spatially explicit metapopulation inhabiting a finite network of patches, and they studied the scale at which the population was adapted. Depending on gene flow and demo-genetic parameters they found that adaptation may be local, at the network scale, or may lead to a mosaic specialisation. They did not, however, specifically address the question of the effect of habitat spatial structures on adaptation.

How does spatial heterogeneity drive the evolution of specialism *vs* generalism? And how does habitat spatial structure determine the level and speed of adaptation? To address these questions, a flexible metapopulation model allowing for different metapopulation structures is developed. Analytical and simulation studies are then used to investigate how ecological trade-off, dispersal range, habitat proportion and habitat spatial structure interplay to influence the evolution of specialisation. We first describe the model and the methods for the model analysis. Then, the results are presented. An invasion analysis is performed and general analytical results are obtained on the singular strategy, its stability and the evolutionary speed. Finally, the role of habitat spatial structure on the pre- (monomorphic population) and post- (when specialists are selected) branching dynamics is investigated.

## Model and Methods

In this section, we first present the model in its more generic form. Then we describe two specific metapopulation structures (hierarchical and lattice) that were used to explore the role of environment composition and spatial organisation on adaptive dynamics. Last, we present the different approaches that were used to analyse the model. Table 1 provides a summary of terms and parameters definitions.

**Table 1:** Definition of the main notations.

Symbols	Description
*General metapopulation*	
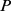	Number of patches
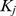	Carrying capacity of patch 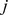
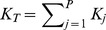	Total carrying capacity of the environment
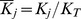	Relative carrying capacity of patch 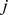
M	Dispersal matrix
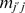	Dispersal rate form patch 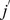 to patch 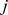
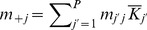	Input connection of patch 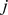
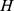	Number of habitats
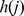	Habitat type of patch 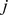
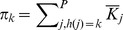	Proportion of habitat 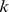 in the environment
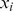	Value of trait 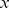 for phenotype 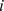 (strategy)
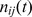	Population size of phenotype 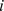 in patch 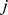 at time 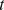
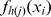	Survival probability of phenotype 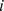 in patch 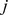
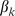	Optimal trait in habitat 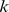
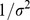	Habitat selectivity
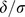	Differentiation between two habitats when only two habitats are present with 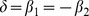
*Hierarchical metapopulation*	
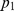	Number of groups
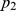	Number of patches per group
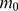	Proportion of the propagules issued from a patch that are deposited in a given patch of another group
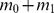	Proportion of propagules that are deposited in another given patch of the same group
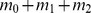	Proportion of propagules that remain in their patch of origin
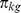	Frequency of habitat 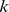 in group 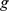
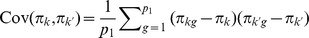	Covariance between habitat group frequencies
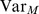	Variance of dispersal rates
*Lattice metapopulation*	
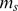	Mean dispersal range
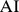	Aggregation index
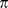	Proportion of habitat 2
*Evolutionary speed*	
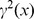	Mutation variance
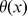	Rate of mutation occurence
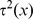	Variability of the offspring distribution of an individual with trait 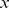
*Post branching simulations*	
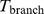	Time to reach the singular strategy
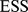	Trait values of the specialists
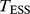	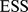 Time taken to reach the
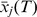	Mean phenotype in patch 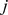 at the end of simulations
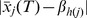	Level of local adaptation at the end of simulations

### Model

The model is based on a discrete-time deterministic description of the population dynamics. It deals with a metapopulation composed of several phenotypes that develop on a spatially heterogeneous environment consisting of a network of 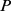 patches. We consider dispersal as a passive process only, *i.e*. there is no habitat choice. This model generalises the Levene's soft selection model [33] to any number of patches interconnected with any set of pairwise dispersal values.

#### Environmental heterogeneity

Two kinds of environmental heterogeneity are accounted for in our model. The first one is related to the patch structure and it acts in the same way on all phenotypes. We shall call it ‘structural heterogeneity’. The second one refers to the habitats, their characteristics, their proportions and their distribution over the different patches. We shall call it ‘habitat heterogeneity’.

Two components account for the structural heterogeneity: the carrying capacity (size) of each patch and the dispersal rates between each pair of patches. In our model, each patch 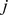 carries a finite and constant number 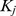 of individuals. We define the relative carrying capacity of patch 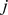 as 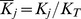, where 
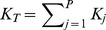
 denotes the total carrying capacity of the environment. Dispersal is heterogeneous so that a propagule dispersed from a patch is deposited in another patch according to a specified dispersal distribution that is not necessarily uniform. The proportion of propagules from patch 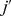 that migrate to patch 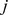 during a life cycle is denoted by 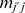. The number of propagules received by patch 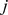 is determined, up to a constant, by the input connection of this patch defined by:
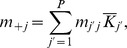



In the general case, no assumptions are required on dispersal rates and the model deals with any metapopulation structure with any set of pairwise dispersal values. Thus it provides a unified framework to handle classical metapopulation as well as spatially explicit models. In addition to this generic environmental structure, two specific types of structural spatial heterogeneity will be given a particular attention. The first one is based on a hierarchical environment structure and the second one on a lattice structure (Section ‘Specific environments’).

The habitat heterogeneity is described as follows: the environment is composed of 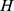 different habitats (or niches) with a single habitat in each patch. The habitat of patch 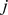 is denoted by 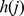. Habitat 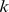 is in proportion 
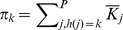
 in the environment. Habitat allocation in space depends on the patch structure and will be explained in Section `Specific environments'.

#### Individuals

Individuals are assumed to be haploid and are classified with respect to their phenotype. They reproduce asexually with non-overlapping generations and the progeny of an individual usually has the same phenotype as its parent. Phenotype 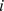 is characterised by the value, or strategy, 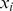 of a continuous trait 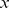. The population size of phenotype 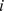 in patch 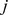 at time 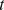 is denoted by 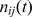. As the total population size is constant, it satisfies
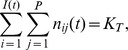
where 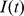 is the largest phenotype index in the metapopulation at time 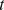.

Given its trait value 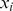 and the habitat encountered in patch 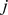 the survival probability of phenotype 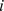 in patch 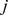 is proportional to 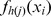, where 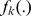 is a fitness function depending on the habitat. In this paper, the function 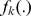 is assumed to be Gaussian [21], so that
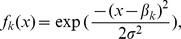
where 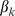 is the optimal trait in habitat 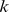 and 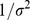 is the habitat selectivity, the same in all habitats. Differences between optimal traits 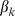 for the different habitats generate a trade-off between survival functions on the habitats: adaptation to a particular habitat causes maladaptation to the others. In particular, consider two habitats 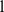 and 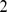 with opposite values of the optimal trait. Then the survival functions of strategy 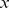 are 
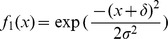
 and 
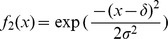
 with 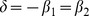. The associated trade-off function, 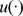, between the survival functions in habitats 1 and 2 is defined by 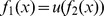. As shown in Débarre & Gandon [25], it satisfies



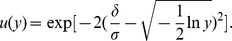



**Figure 1:**
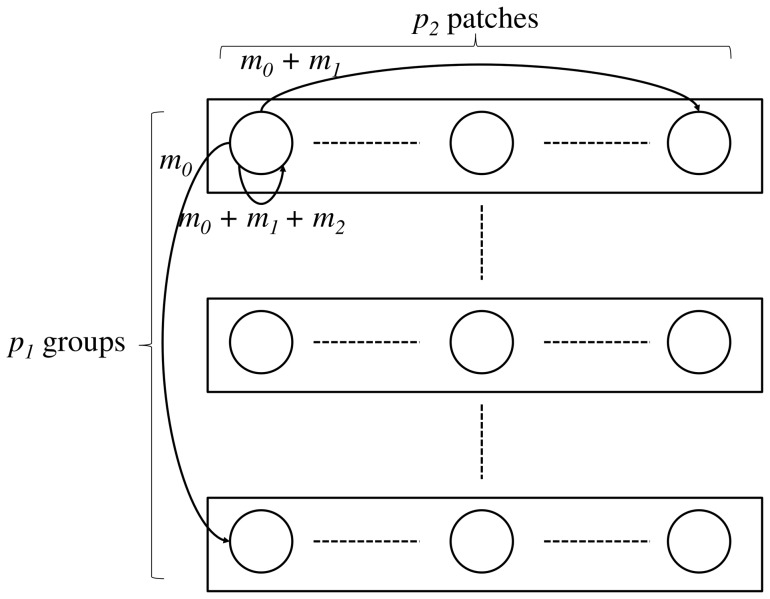
Hierarchical environment structure. 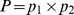
 patches are distributed among 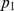 groups of 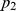 patches. 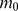: dispersal rate between patches that belong to different groups, 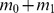: dispersal rate between patches that belong to the same group, 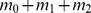: intra-patch dispersal rate.

The differentiation between the two habitats is quantified by 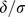. When 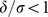, the trade-off is weak. When 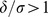, the trade-off is strong. When 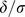 is close to one, the trade-off is very sensitive to the phenotype value 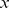 ([25], Figure 1c).

#### Metapopulation dynamics

The demography of the metapopulation is modelled using deterministic discrete-time equations. The model is based on the life cycle of individuals that involves the following sequence of events: reproduction, dispersal, selection and regulation. Reproduction rates are assumed to be constant among habitats and phenotypes, so we only present in detail the dispersal, selection and regulation phases.

During dispersal, a proportion 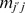 of propagules produced in patch 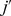 is deposited on patch 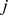. So, up to a constant, the number of propagules of phenotype 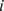 that are deposited on patch 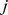 is equal to
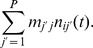



In each patch, new individuals are subject to a selection process with a survival probability of phenotype 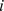 in patch 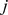 proportional to 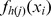. Thus, in patch 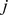, the number of surviving individuals of phenotype 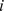 is proportional to:
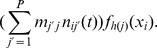



After the selection phase, a non-selective local regulation for space makes the population size of phenotype 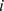 on patch 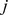 at time 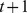 equal to
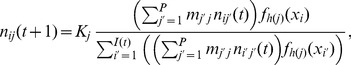
(1)where the sum is performed over all phenotypes 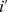 present in the metapopulation at time 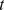.

### Specific environments

Two specific environments will be studied in detail with the model described above. The first one is a hierarchical environment with three nested levels: patch, group of patches and whole set of patches. It describes space as a partition between groups made of mutually neighbour patches. The second one is a lattice environment with dispersal rates based on euclidean distance. In this case, space is explicit and two-dimensional.

The hierarchical and lattice environments are two different and complementary ways of representing spatial relationships between patches. So we will study the influence of habitat distribution in both environments in parallel. In addition, the hierarchical environment will allow us to go further in analytical developments, while the lattice will be used to explore the post-branching dynamics by simulation.

#### Hierarchical environment. *Structural heterogeneity*

The hierarchical environment consists of a network composed of 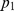 groups of 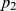 patches of the same carrying capacity (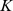), so that 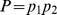 (Figure 1). Three dispersal rates 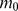, 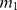 and 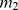 are defined: 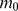 is the proportion of the propagules issued from a patch that are deposited in a given patch of another group, 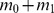 is the proportion of propagules that are deposited in another given patch of the same group, 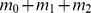 is the proportion of propagules that remain in their patch of origin. Each propagule lands in one patch so that 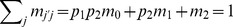. This parametrisation leads to the dispersal matrix
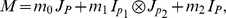
where 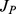 is the 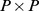 matrix of ones, 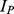 is the identity matrix of size 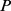 and 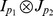 denotes the block-diagonal matrix with diagonal matrices 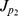. The symbol 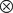 denotes the Kronecker product between matrices. Note that when 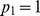 or 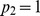 the hierarchical environment corresponds to the Deakin's model [34].

#### *Habitat heterogeneity*

The 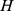 habitats are dispatched among the patches in proportions 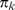, for 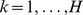 and their spatial distribution is defined by the frequencies 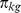 of habitat 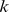 in group 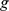. It is also characterised by the covariances
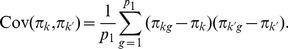



If the proportion of habitat 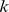 or 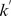 is constant across the groups, then the covariance is zero. In particular, this is the case for all pairs of habitats if they are spread homogeneously across the groups. Otherwise, if two habitats are distributed identically across the same groups, their covariance is positive. But if they are aggregated in distinct groups, their covariance is negative. In particular it reaches the value 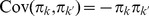 when each 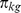 is either zero or one, that is, when each group contains only one habitat.

#### Lattice environment. *Structural heterogeneity*

The lattice environment consists of a continuous square area 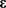 partitioned into a regular lattice of square patches. Dispersal is assumed to be isotropic and to decrease exponentially with distance. More precisely, the proportion of propagules dispersed from a given source point 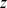 and arriving at a given reception point 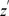 is given by the individual dispersal function
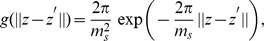
where 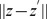 is the euclidean distance between 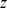 and 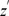, and 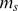 is the average dispersal range ([35], Appendix C). The between-patch dispersal proportions are deduced by integration according to the formula



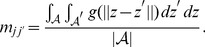
(2)When using Equation (2), the integration is defined over the pairs of points that belong to the domains 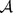 and 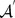 of patches 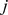 and 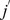, respectively, and 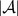 is the area of patch 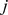. The implicit assumption is that the population mixes perfectly in each patch.

The lattice environment was studied by numerical calculation and by simulation. It was fixed to a size of 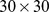 length units, resulting in 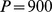 contiguous square patches of area equal to one. To avoid border effects, 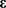 was considered as a torus in the calculations of dispersal rates and habitat aggregation (see after). The dispersal rates 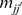 in Equation (2) were computed using the CaliFloPP algorithm [36] on the 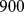 patches.

#### *Habitat heterogeneity*

**Figure 2:**
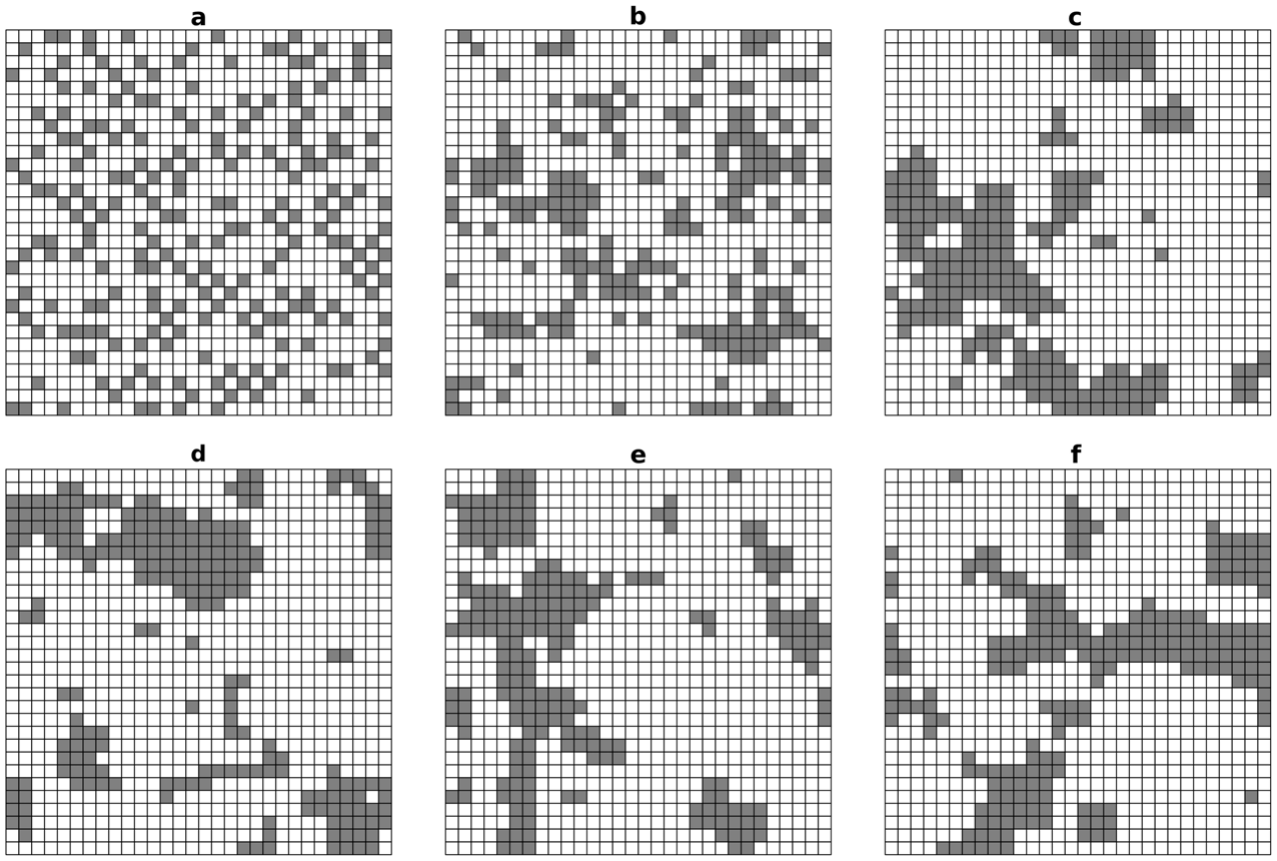
Distribution of habitats in the lattice environment. Distribution of habitats in the lattice environment. Habitat 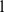 (white) and habitat 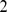 (grey) represent 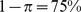 and 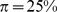 of the environment, respectively. Top row: increasing aggregation index (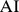); bottom row: three pseudo-random habitat allocations. a: 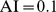; b: 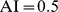; c, d, e and f: 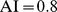.

In the lattice environment, the spatial distribution of habitats is characterised by the proportions 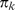 and by an aggregation index, 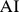 [37]. This index varies between 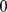, when two patches sharing the same habitat are never neighbours, and 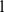, when patches sharing the same habitat are as clustered as possible (Figure 2, top line). In the numerical calculations and in the simulations, two habitats (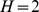, with 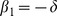 and 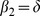) in proportions 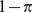 and 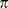 were allocated to the 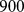 patches. Without loss of generality, we only considered patterns that satisfied 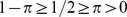. For a particular combination of 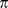 and 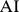, several habitat allocations were considered (Figure 2, bottom line).

### Model analysis

The model described above was analysed in three stages. First, we carried out an invasion analysis. Under the assumption that the metapopulation is monomorphic, the condition was established for the metapopulation to evolve towards either one generalist or several specialists (branching criterion). We derived a mathematical expression of the branching criterion for a general environmental structure. Then this criterion was simplified in the hierarchical case and it was explored numerically in the lattice case. Second, we studied the evolutionary speed to reach the branching point in a monomorphic metapopulation. Here again a general environmental structure was first considered before applying the results to the hierarchical environment. In the lattice environment, the evolutionary speed was analysed using a simulation model. Third, the post-branching evolution of the specialists was studied in the lattice environment by a simulation approach based on Equation (1).

#### Invasion analysis

**Figure 3:**
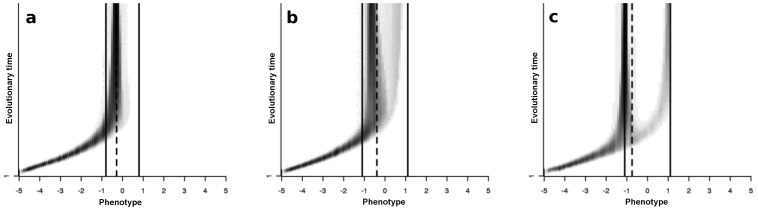
Examples of evolutionary trajectories simulated from Equation (1) on the lattice environment. The branching criterion (Equation (4)) is equal to 0.6 (a), 1.07 (b) and 7.5 (c). When it is lower than one, the generalist strategy is stable (a). Otherwise, the metapopulation splits into two sub-populations of specialists (b and c). Dashed line: theoretical singular strategy (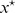), solid lines: habitat optima (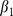 and 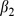). Other parameters are: a: 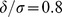 (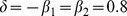 and 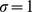), 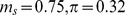, 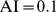; b: 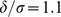 (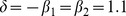 and 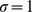), 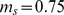, 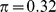, 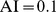; c: 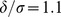 (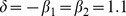 and 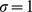), 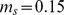, 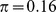, 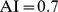.

We considered a standard and simplified framework whereby the metapopulation is monomorphic and evolves through episodic mutations of small amplitude. According to the theory of invasion analysis [21], the metapopulation evolves towards the ‘evolutionarily singular strategy’ characterised by the trait value 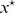, if 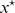 is an attractor (i.e. 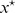 is ‘convergence-stable’). The singular strategy corresponds to a generalist phenotype. If 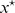 is an ‘evolutionarily stable strategy’ (ESS), the population remains monomorphic and the environment thus selects for a generalist phenotype. Otherwise, i.e. 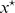 is convergence-stable but not an ESS, 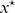 is a branching point. In this latter case, some phenotypes on opposite sides of 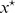 can coexist and the population becomes polymorphic, *i.e*. the environment selects for specialist phenotypes. Figure 3 gives some examples of evolutionary trajectories in both cases.

We applied the theory of invasion analysis to our setting using a matrix model based on Equation (1) ([38], chapter 4) to describe the demography of a mutant with trait 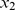 in a resident population with trait 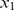. As long as the phenotype 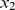 is rare, 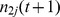 satisfies:
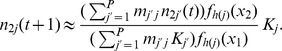



The vector 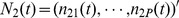 (prime denotes transposition) of the numbers of mutant individuals in the different patches at time 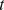 satisfies thus the matrix equation 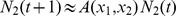, where 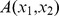 is the 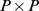 projection matrix [38]. The element of 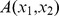 in row 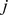 and column 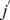 gives the number of mutant individuals in patch 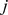 resulting from a single mutant individual in patch 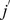 and is equal to
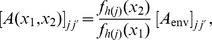
where the matrix 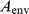 depends on the environment structural heterogeneity only and is defined by



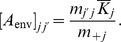



Following Durinx *et al*. [39], the mutant invasion fitness function is defined by 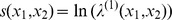, where 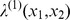 is the dominant eigenvalue of 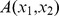. As mutation steps are small, the first order approximation of the invasion fitness 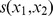 gives
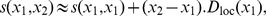
where 
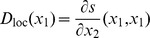
 is the local fitness gradient [21]. Its sign determines the direction of selection: if 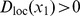, then only mutants with 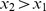 can invade, whereas if 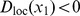, then this is possible for mutants with 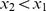 only. The trait value 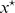 for which the local fitness gradient is zero, *i.e*. 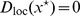, corresponds to the ‘singular strategy’. It is convergence stable if the resident population evolves towards 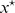, which occurs if the derivative of the local fitness gradient 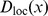 is negative at 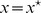 [21]. Otherwise 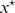 is an evolutionary repeller. The evolutionarily singular strategy 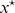 depends on the eigenvectors of 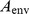. The eigenvalues of 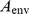 are denoted in decreasing order by 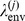 for 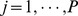 and the corresponding left and right eigenvectors are denoted by 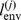 and 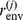. In the Section ‘Results’, we derive the evolutionarily singular strategy and we characterise its stability by an analytical expression of the branching criterion. After deducing the analytical expression of the branching criterion, we study it in more detail in the hierarchical and lattice environments. Technical support for the invasion analysis is given in Appendix S1.

For the hierarchical environment, analytical results are presented and interpreted. For the lattice environment, the branching criterion had to be computed numerically and so three global sensitivity analyses were performed at different values of the dispersal range 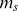 [40]. The three values of 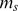 were 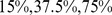 of the environment range. The same three input factors were varied in each sensitivity analysis: (i) the proportion 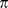 of habitat 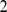; (ii) the aggregation index 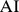 and (iii) the habitat differentiation 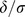. The proportion 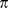 was varied at ten levels in the range 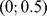. The 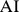 index was varied at eight levels in the range 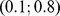. The habitat differentiation 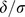 was varied in the range 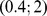 by fixing 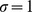. A simulated annealing algorithm [41] was used to generate environments with controlled values of 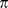 and 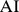 (see Figure 2, top line). For each combination of 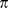 and 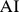, up to 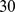 habitat allocation replicates were randomly generated (Figure 2, bottom line).

In the Section ‘Results’, sensitivity indices are decomposed into main effects (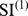), two-factor interactions (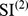) and the triple interaction (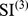) of the three input factors 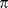, 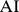 and 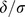. In addition, the total effect (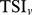) of a factor is defined as the sum of its main effect and of the interactions involving that factor. Each index is the proportion of the branching criterion variability explained by a given factorial term, so that it varies between 0 (no effect) and 1 (maximum effect). We used the definition of sensitivity indices based on the Sobol' decomposition [42] and applied it to the branching criterion considered as a function of the three input factors. The sensitivity indices were estimated by the metamodelling technique which consists in approximating the function under study by a polynomial chaos expansion [43].

#### Evolutionary speed

The evolutionary speed of the resident phenotype trait 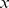 in a monomorphic population is defined as the derivative 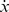 of 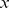 on time at a large scale. It can be approximated by the canonical equation of adaptive dynamics [44], [45], which is based on asymptotics with three nested time scales. At the finest time scale, time is discrete and the dynamical model (Equation (1)) applies to each generation. At medium time scale, the number of generations per time unit tends to infinity. Time appears as continuous but phenotype trait evolution still appears as discrete with a series of monomorphic resident metapopulations each identified by its unique phenotype. At large time scale, a large number of small mutations occur at each time unit, so that the phenotype trait evolution appears as a continuous and derivable process. More precise mathematical details are given in Appendix S2.

In order to study the evolutionary speed up to the singular strategy in our setting, the population was still assumed to remain monomorphic and the mutations were assumed to have a small amplitude, just as for the invasion analysis. In addition, each mutation was assumed to alter the phenotype trait with a small variance 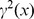 and to occur according to a continuous-time Poisson process with rate 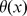. The stochasticity that affects the demography of the mutant when it is still rare was taken into account through a parameter 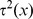 that quantifies the variability of the offspring distribution of an individual with trait 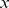 (Appendix S2). Under these assumptions, the canonical equation of adaptive dynamics was first obtained in the general case. It was then applied analytically to the hierarchical environment. For the lattice environment, the time to reach the singular strategy was estimated using the simulation model described in Section ‘Post-branching evolution’.

#### Post-branching evolution

In our framework, the main assumption required for analytical developments is that of a monomorphic population. As a consequence, it is not possible to obtain analytical results when several specialists are present in the metapopulation. In order to study the effects of environmental heterogeneity on the specialist strategies after branching, we had to relax the monomorphic population assumption. For this, we developed a simulation model, based on Equation (1), representing the evolution of a metapopulation whatever its phenotypic composition.

The model assumptions regarding the environment and the dispersal function were those of the lattice environment (see Section ‘Lattice environment’). The patch carrying capacity was set to 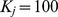 for all patches. The continuous trait 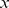 varied between 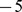 and 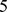 and this range was discretised into 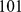 phenotypes. The simulations started with a monomorphic population with trait value 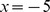. At each time step, mutants were generated from an existing phenotype provided its local population size was over a threshold of 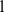. New mutants were generated according to a Gaussian perturbation centred on the pre-existing trait value with a variance of 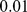. There was no genetic drift. The multitype metapopulation dynamics followed Equation (1) and were simulated over 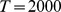 time steps.

**Figure 4:**
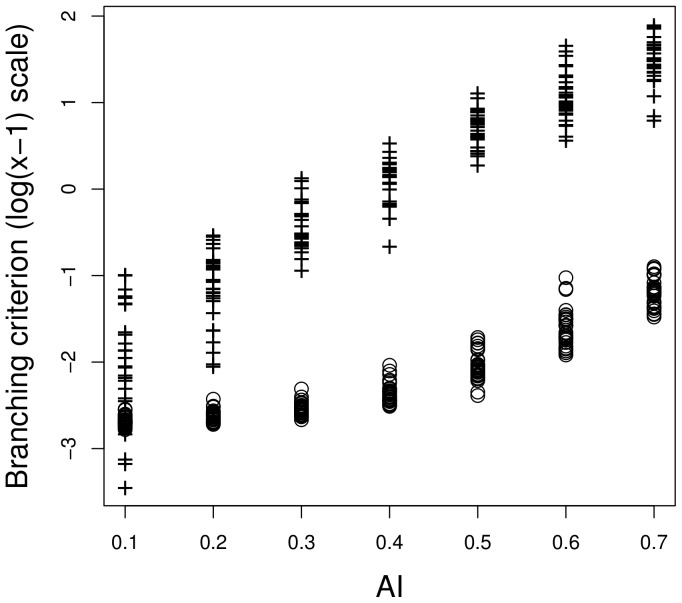
Value of the branching criterion against the aggregation index (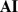). Open circles, situation A (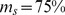, 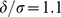 (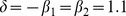 and 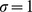) and 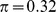); pluses, situation B (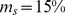, 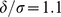 (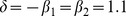 and 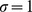) and 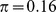). The lattice environment was used.

Two situations were selected to study the post-branching evolution. For each situation, the values of the parameters 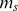, 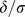 and 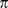 were chosen so that a branching occurs whatever the aggregation index. They were set to 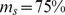 and 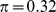 in situation A and to 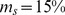 and 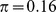 in situation B. In both situations habitat differentiation was equal to 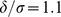 (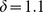 and 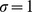) and the aggregation index 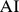 was varied by 0.1 in the range 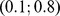. In situation A, the large dispersal range reduced the influence of the spatial structure and the branching criterion was little sensitive to the aggregation index (Figure 4). In situation B, the dispersal range was smaller, so that there was a wide range of branching criterion values when the aggregation index varied (Figure 4). Two contrasted examples of evolutionary trajectories when the branching criterion was close to 1 or much more greater than 1 are shown in Figure 3a and b, respectively.

For each situation and each 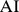 value, 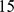 replicates were conducted, each on a different environment pattern generated at random as described in Section ‘Lattice environment’. Before branching, we estimated the time 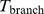 taken to reach the singular strategy. After branching, the metapopulation was considered as a mixture of two specialists. Simulations were then compared through three criteria: (i) the trait values of the specialists (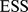), (ii) the time taken to reach these values (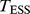) and (iii) the specialist's phenotypic variance at final time 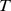. In addition, the level of adaptation of local populations was computed at the end of the evolution process within each patch. It was defined as the mismatch between the mean phenotype 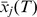 and the optimal phenotype 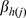: 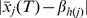.

Technical details on how these criteria were calculated are given in Appendix S4 and Figure S1. The consistency between the analytical and the simulation approaches is discussed in Appendix S5 and Figure S2.

## Results

We first present the results of the invasion analysis and the computation of the evolutionary speed in the general situation. This leads to the mathematical expression of the singular strategy and of the branching criterion. Then, we study the pre- (when the population is monomorphic) and post- (when specialists are selected) branching evolutionary dynamics by examining how dispersal and habitat heterogeneity influence the selection for specialists, the evolutionary speed and the adaptive dynamics of specialists when branching occurs.

### Invasion analysis and evolutionary speed: General case

#### Singular strategy

We show in Appendix S1 that a monomorphic population first evolves towards a unique singular strategy 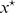 equal to
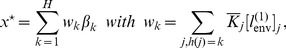
(3)where the eigenvectors 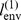 and 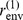 are normalised so that 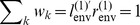. Thus the singular strategy is the barycentre of habitat optima: it corresponds to a generalist phenotype. Note that in this model, the singular strategy can always be reached by gradual evolution, *i.e*. convergence stability always occurs (Appendix S1).

When dispersal rates are symmetric, *i.e*. 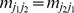, the 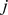
th coordinate of the eigenvector 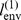 is equal to the input connection 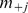 of patch 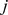. The singular strategy is then equal to
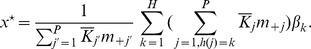



In other words, the singular strategy is an average of the habitat phenotypic optima 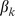, weighted by an increasing function of the relative carrying capacities and input connections of the patches: small or isolated patches have a smaller influence on the generalist phenotype.

If all the patches have the same carrying capacity and the same input connection, the singular strategy is equal to
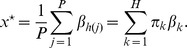



In this case, the weights are equal to the habitat proportions and the spatial allocation of the different habitats has no impact on the singular strategy (see also [25], equation (17)).

#### Branching criterion

In Appendix S1 we show that the singular strategy 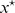 is an ESS and thus that the environment selects for a generalist phenotype if and only if
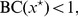
(4)where the branching criterion 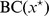 is defined by



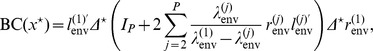
where prime denotes transposition. In this expression, 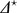 is the 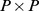 diagonal matrix whose diagonal elements are given by 
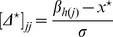
. The left and right eigenvectors of 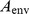 are normalised so that 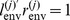, 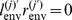 for 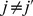.

The branching criterion depends on the patch structure and on the habitat phenotypic optima. The expression is too complex to be interpreted directly, but it opens the way to a better understanding of how environment heterogeneity and structure influence the stability of the singular strategy. This will be illustrated in the following sections.

#### Evolutionary speed

Under the notations and assumptions of Section ‘Evolutionary speed’ in ‘Models and methods’, the canonical equation of adaptive dynamics is (Appendix S2)
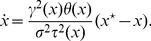
(5)


Thus the speed increases with the mutation amplitude 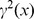, the mutation rate 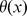 and the habitat selectivity 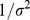, while it decreases with the offspring variability 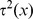 because of an increasing risk of loss by drift. It also decreases progressively when the population comes closer to the singular strategy. Provided 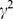, 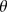 and 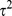 do not depend on 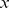, the solution to Equation (5) satisfies:
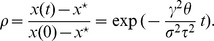



Thus the distance 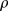 to the singular strategy 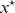 relatively to the initial distance 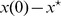 decreases exponentially. Conversely, the time taken by trait 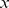 to arrive within the relative distance 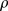 is
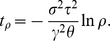



Parameters 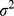, 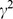 and 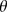 do not depend on structural and habitat heterogeneities. Therefore, structural and habitat heterogeneities influence the speed of adaptation only though parameter 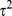, the offspring variability (Appendix S2 and Section ‘Evolutionary speed’).

### Effect of environmental heterogeneity on specialisation: Pre-branching dynamics

#### Branching condition when dispersal is homogeneous

When dispersal is homogeneous, *i.e*. the metapopulation is not spatially structured, the condition for evolutionary stability in Equation (4) is:
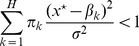
(6)


(Appendix S3). This result has already been established by Geritz *et al*. ([21], Appendix 2). Considering that the singular strategy 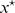 is a weighted mean of the habitat optima (Equation (3)), the left-hand side of inequality (6) can be considered as a variance ratio measuring global habitat differentiation. When dispersal is homogeneous, this global habitat differentiation is sufficient to determine whether branching will occur or not: only the non-spatial components (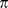 and the 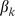) of the habitat heterogeneity are involved.

If there are two habitats in proportions 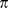 and 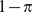 with 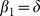 and 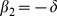, Equation (6) becomes:
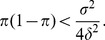
(7)


This simple inequality shows that specialisation is facilitated when the habitats are highly differentiated (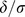 is large). Equation (7) also shows that both habitats must exist in sufficient proportions for specialists to emerge. Otherwise, evolution leads to a single generalist poorly adapted to the habitat with the lowest proportion, according to Equation (3).

#### Branching condition when dispersal is hierarchical

We now consider the hierarchical environment described in Section ‘Hierarchical environment’ (Figure 1). The condition for evolutionary stability in Equation (4) becomes (Appendix S3):
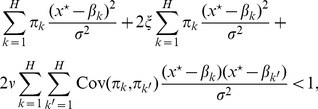
(8)where 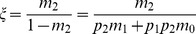 and 
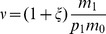
. The first term on the left-hand side is the global habitat differentiation of Equation (6). The hierarchical structure adds the next two terms which are positive (Appendix S3), indicating that heterogeneity in dispersal makes specialisation easier.

The second term is related to the effect of purely local dispersal. Indeed it is the product between the global habitat differentiation and parameter 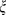 that quantifies patch isolation in terms of propagule exchanges. Parameter 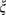 varies between 0, when the within-group dispersal is homogeneous, and 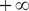, when the dispersal is purely local inside the patches. This term indicates that a low habitat differentiation (weak trade-off) can be compensated by a high level of patch isolation with regard to the emergence of specialists.

Existence of a group structure appears explicitly in the third term of Equation (8). It is the product between a measure of habitat aggregation across groups and parameter 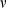, which is a synthetic measure of patch and group isolation with respect to dispersal. When the habitats are distributed homogeneously between groups, this third term vanishes since 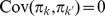 for all 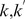 (see Section ‘Hierarchical environment’). On the contrary, when groups are unbalanced with respect to habitat proportions, the third term increases up to the extreme situation in which each group contains only one habitat. Increasing habitat differentiation between groups increases the third term of Equation (8) and makes specialisation easier. As a consequence, generalist strategies are favoured when habitat composition of the groups is homogeneous, that is when there is no habitat clusters in the environment.

Consider now the situation when two habitats are present in proportions 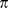 and 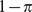 with 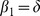 and 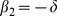. If each group of patches is composed of one habitat only, Equation (8) becomes:
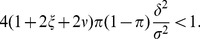
(9)


This relation shows that dispersal, habitat proportion and habitat differentiation affect the branching criterion in a multiplicative way (see also the sensitivity analysis on the lattice environment). Specialisation is favoured when habitat proportion is balanced (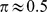) and when habitat differentiation is high. In addition, limiting dispersal, either by increasing patch (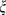) or group (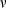) isolation, facilitates branching. Conversely, if habitats are mixed within groups so that each group is composed of both habitats in proportions 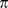 and 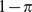, Equation (8) becomes:
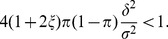
(10)


The comparison of Equations (9) and (10) clearly shows that mixing habitats within groups favours the generalist over the specialists.

To summarise, Equation (8) allows to separate the effects of global (first term), local (second term) and spatial (third term) components of habitat heterogeneity on the branching criterion.

#### Interactions between dispersal, habitat proportion and habitat allocation

The weight of each term in Equation (8) depends on the dispersal rates. For instance, the effect of the group composition measured by 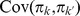 is greater for stronger group isolation as measured by 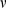. In addition, habitat allocation and proportion interact strongly. The range of variation of 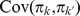 is maximal when 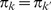 and overall the spatial component of the branching criterion decreases when a habitat is increasingly present in the environment (Equation (8)). This means that when the proportion of the major habitat increases in the environment, habitat allocation in space has less and less influence on the branching criterion.

**Figure 5:**
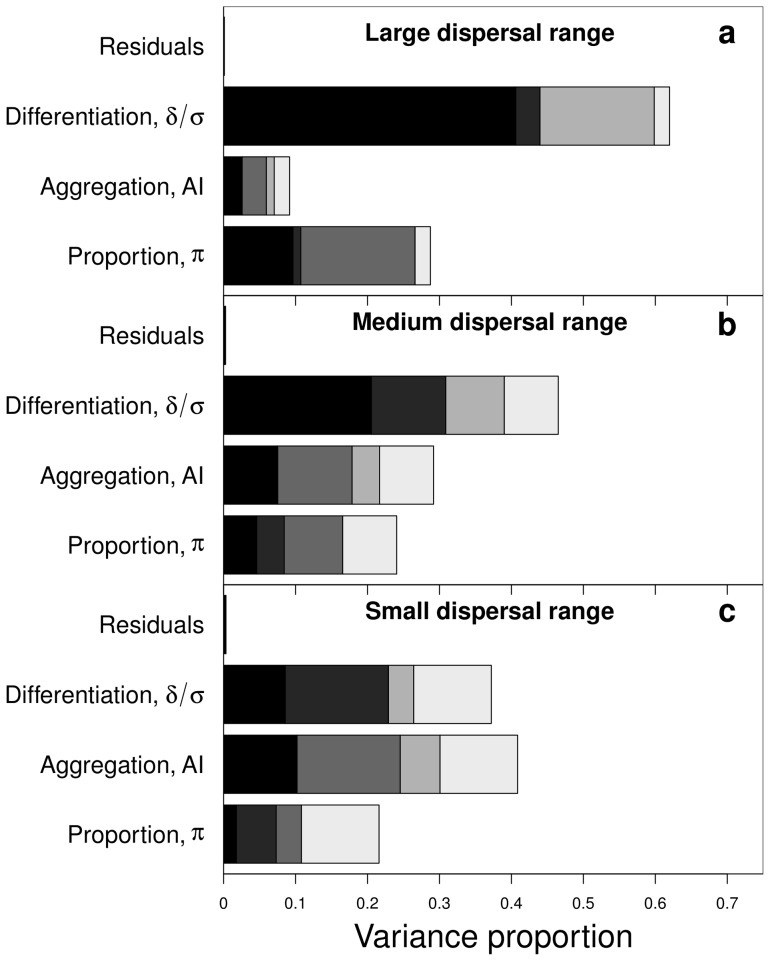
Sensitivity of the branching criterion given in Equation (4) to habitat differentiation (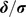), habitat aggregation index (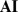) and habitat proportion (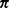). a: large dispersal range (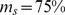); b: medium dispersal range (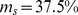) and c: small dispersal range (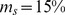). Black bar: main effect (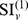); from the darkest to the lightest grey bars: interaction 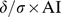 and 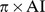; interaction 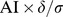 and 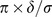; interaction 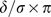 and 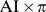; and triple interaction. The total bar length indicates the total effect (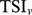). The lattice environment was used.

These analytical results on the hierarchical environment are consistent with the sensitivity analyses performed on the lattice environment (Figure 5). The sensitivity indices showed that more than 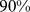 of the branching criterion variability is explained by non-spatial components when dispersal range is large (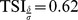 and 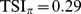 when 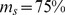) whereas this proportion drops to 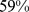 for a small dispersal range (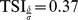 and 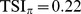 when 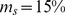). Note that the variability of the branching criterion is mainly explained by interactions between factors, including the third order interaction, especially when the dispersal range is low (Figure 5).

#### Effect of habitat spatial distribution

**Figure 6:**
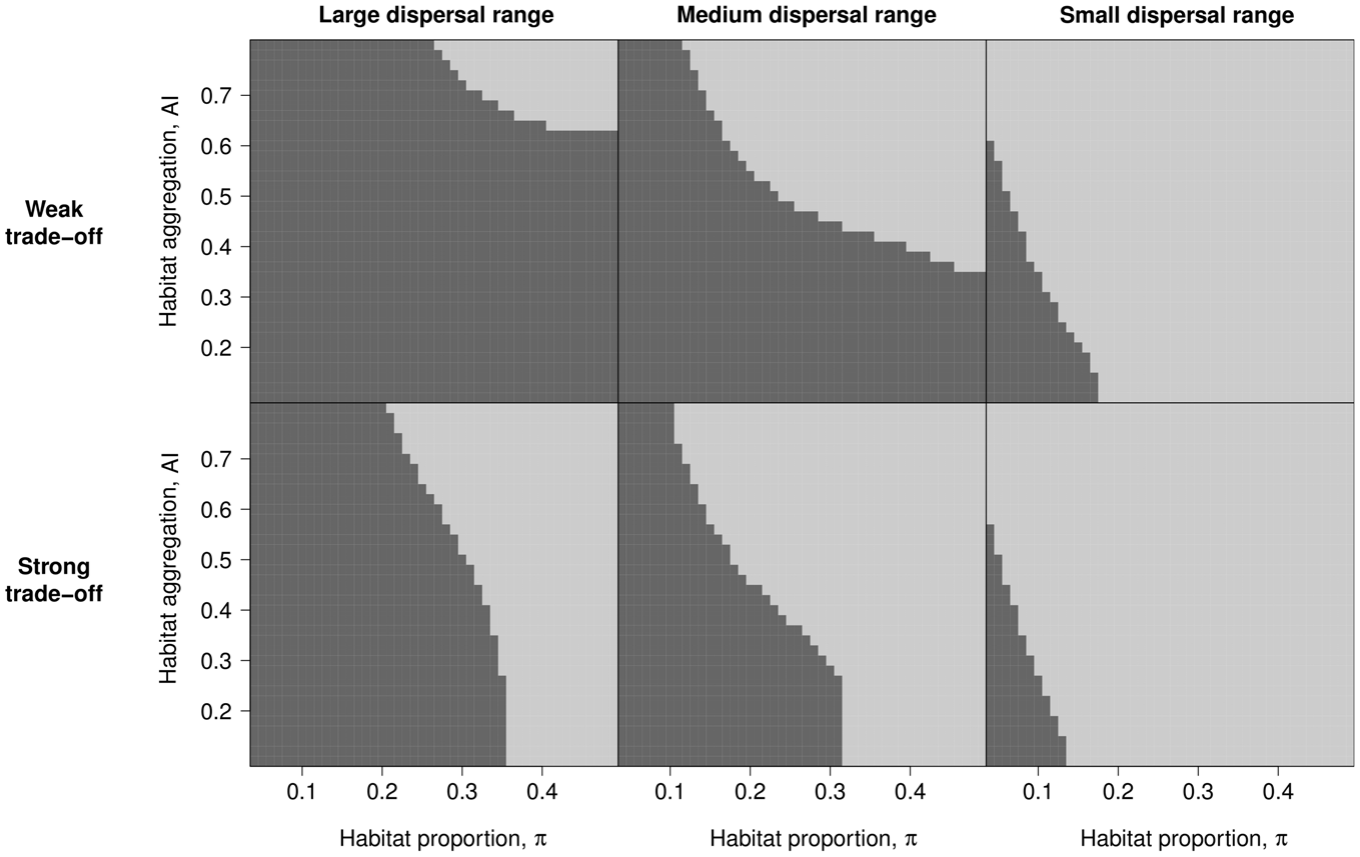
Stability of the generalist strategy against habitat 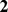 proportion (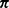) and habitat aggregation index (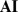). The generalist strategy is stable when the branching criterion (Equation (4)) is lower than 1 (dark grey) and unstable when it is greater than 1 (light grey). In the top row the trade-off is weak (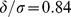, 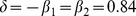 and 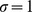), in the bottom row the trade-off is strong (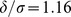, 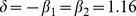 and 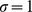). Left column, dispersal range 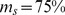; middle column: dispersal range 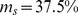 and right column: dispersal range 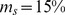. The lattice environment was used.

Here, we specifically investigate the issue of habitat allocation in space. Figure 6 maps the stability of the singular generalist strategy in the lattice environment as a function of 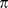 and 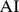, for different levels of habitat differentiation and dispersal range. Figure 6 shows that an increase in the proportion 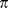 of the minor habitat as well as an increase in habitat aggregation 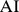 make selection for specialist phenotypes easier. If the dispersal range 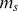 decreases, or if habitat differentiation 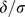 increases, branching occurs for lower values of 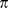 and 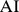. In case of a strong trade-off (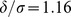), this determines a threshold for 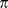 above which specialisation is observed whatever the aggregation level. The threshold value depends on the dispersal range (Figure 6, bottom line). In addition, when the dispersal range is small (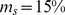) and the aggregation is high (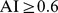), specialisation is observed whatever the value of 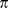 and 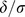.

The effect of habitat allocation can be further explored with the hierarchical environment. Consider that the parameters 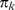, 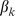, 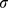, 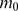, 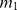 and 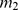 are fixed and that the within-group habitat frequencies 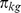 are controlled. Then the global and local components of the branching criterion in Equation (8) are fixed and the branching criterion is minimal when the spatial component is zero. This occurs when 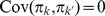, that is, when 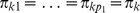 for 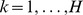. This establishes an optimality property in the hierarchical case: allocating habitats to patches so that all the groups have the same habitat composition is optimal for limiting specialisation.

#### Evolutionary speed

As it was explained in Section ‘Evolutionary speed’ in ‘Results’, structural and habitat heterogeneities influence the speed of adaptation through parameter 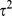 only. In the case of a hierarchical environment, an analytical expression for parameter 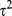 can be provided. Since all patches have the same carrying capacity and since dispersal rates are symmetric, 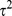 is equal to (Appendix S2):
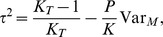
where 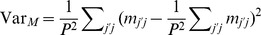, that is, 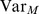 denotes the variance of dispersal rates. Remarkably, in that case 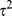 does not depend on the allocation of habitats in space. It only depends on the dispersal rates through their variance. When dispersal is homogeneous (*i.e*. the metapopulation is not spatially structured, 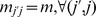), 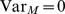 and the time to reach the singular strategy is maximal. As the variance of the dispersal rates increases, the population evolves more rapidly up to the singular strategy (Equation (5)). Thus, adding heterogeneity in dispersal (*i.e*. increasing 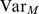) makes specialisation easier and faster.

This effect is found again in the lattice environment, in which the time to reach the singular strategy was estimated by simulations (Figure S3). In situation A (large dispersal range thus low structural heterogeneity) the singular strategy was globally reached slower than in situation B (low dispersal range thus high structural heterogeneity). In addition, in situation B, the singular strategy was reached significantly faster when habitats were aggregated.

### Effect of environmental heterogeneity on the adaptive dynamics of specialists: post-branching dynamics

We now only consider situations in which the singular strategy is unstable, *i.e*. the metapopulation splits into two phenotypic morphs of habitat specialists, and we examine the effect of habitat spatial structures on the evolutionary dynamics of the specialist phenotypic morphs. The monitoring of the specialist's phenotypic variance revealed a first diversification phase during which the phenotypic variance increased then a selection phase during which the phenotypic variance decreased until it was stable (Figure 3 and Figure S4). The phenotypic variance reached its maximum at the singular strategy. In the following we only discuss the effect of habitat aggregation on the phenotypic morphs but the other parameters could also have an influence. For example, Figure S4 shows that the decrease in both 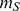 and 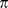 decreased phenotypic variance of the specialist morphs.

**Figure 7:**
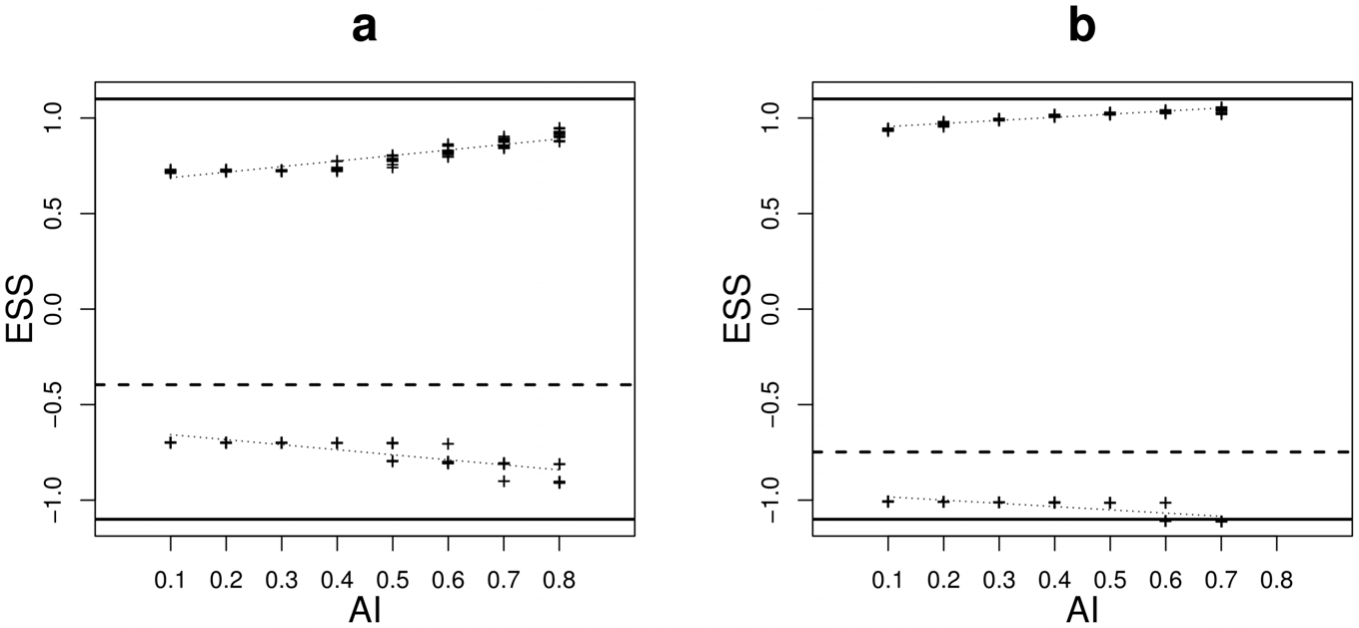
Specialist trait values at the equilibrium (ESS) plotted against the aggregation level of habitat 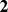 (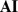) for situation A (a) and situation B (b). The relationship between ESS values and 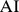 was tested by linear regression (dotted line). Dashed line, singular strategy; solid lines, habitat optima. Other parameters are for a: 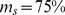, 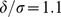 (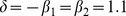 and 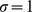) and 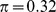; for b: 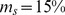, 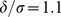 (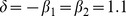 and 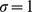) and 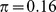. The lattice environment was used.

**Figure 8:**
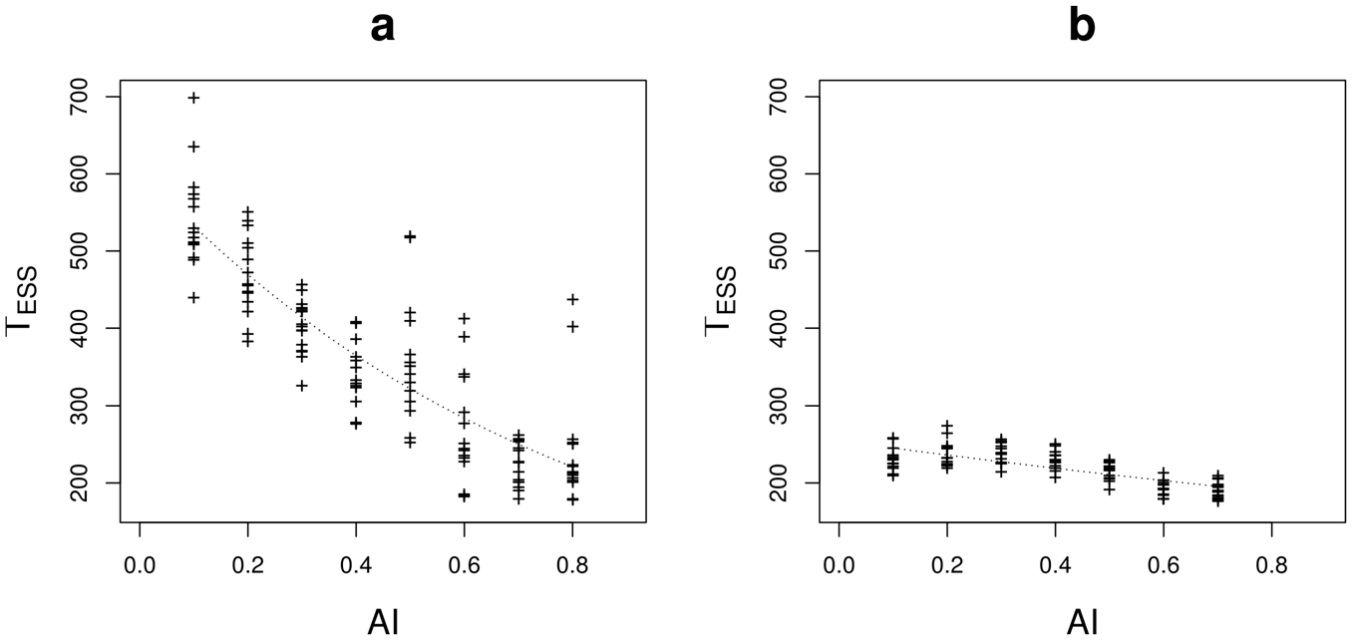
Time to reach the ESSs (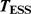) plotted against the aggregation level of habitat 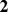 (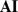) for situation A (a) and situation B (b). The relationship between 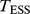 and 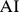 was tested by a GLM with Poisson distributed errors (dotted line). Other parameters are for a: 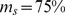, 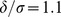 (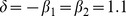 and 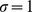) and 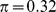; for b: 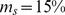, 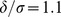 (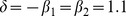 and 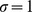) and 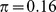. The lattice environment was used.

The simulations showed that specialists adaptation to each habitat was higher (Figure 7) and that the specialist's phenotypic variability was lower (Figure S4) when habitats were more aggregated (higher 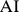). Moreover, specialist phenotypic morphs evolved more rapidly to their stable strategies when habitats were aggregated (Figure 8). Thus, specialists evolved faster and towards populations that were better adapted and more homogeneous when habitats were aggregated.

The adaptation pattern across the environment at the end of the simulations showed that populations at the centre of habitat aggregates had a high local adaptation level whereas populations in isolated patches or developing at the edges of patch aggregates tended to be poorly adapted (Figure S5). These differences in local adaptation were not explained by the persistence of a generalist in the edges but rather by the coexistence of the two specialist phenotypic morphs in the same patch due to the migration-selection balance [46]. This was observed in all simulations satisfying the condition for branching.

The branching criterion characterises the stability of the singular strategy but it is also strongly related to other characteristics of the adaptive dynamics of the specialists after branching. Figure 4 shows the branching criterion values in situations A and B. High values of the branching criterion are related to high evolutionary speeds and high levels of specialists adaptation. In addition, the effects of environmental heterogeneity on the global evolutionary dynamics are greater when the branching criterion is close to 1.

## Discussion

In this article, we addressed two questions related to the dynamics of adaptation from a theoretical point of view: How does spatial heterogeneity drive the evolution of specialism *vs* generalism? And how does habitat spatial structure determine the level and speed of adaptation? To this aim, we developed a model that describes the phenotypic changes occurring in a metapopulation under soft selection and, with this model, we studied the consequences of spatial heterogeneity on the dynamics of adaptation of a population that lives on a finite network of patches interconnected via passive dispersal. Compared to other published models in adaptive dynamics, no assumption is needed here on dispersal rates, which makes our framework very general. By analysing the model through analytical as well as simulation methods, we were thus able to extend classical results to any spatial metapopulation: (i) we provided the singular strategy, (ii) we characterised its stability and (iii) we provided the evolutionary speed. We also (iv) studied the effects of spatial heterogeneity on the specialist strategies.

The singular strategy corresponds to a generalist phenotype since it represents a balanced strategy with respect to habitat frequencies, spatial distribution and optima. When dispersal rates are symmetric and patches have the same input connection, the singular strategy is a function of habitat proportions and optima only and is independent of the spatial distribution of habitats in the environment. This result has already been established by several authors, such as Geritz *et al*. [21] or Débarre & Gandon [25]. Equation (8) incorporates this result but extends it to any dispersal structure. In a general case, spatial configuration may indeed influence the singular strategy. For instance, small or isolated patches have a smaller weight on the generalist phenotype evolution. Although this had been pointed out in the case of the dynamics and persistence of a metapopulation by Ovaskainen & Hanski [29] and discussed by Hanski *et al*. [32] from an evolutionary perspective, the role of individual habitat patches had not previously been demonstrated in an adaptive dynamics approach.

The stability of the singular strategy, characterised by the value of the branching criterion, determines whether the population remains monomorphic with a generalist phenotype or splits into two specialised phenotypic morphs. We found that specialisation is facilitated when habitats are highly differentiated and that habitats must exist in sufficient proportions for specialists to emerge. However, a low habitat proportion or differentiation can be compensated by limiting propagule exchanges between habitats either by hindering dispersal or by aggregating habitats. In addition, the components of environmental heterogeneity interact strongly to determine the value of the branching criterion. For example, when the proportion of a particular habitat increases in the environment, the effect of habitat allocation in space on the branching criterion decreases.

The first models that investigated the respective roles of environmental heterogeneity and dispersal rate on the stability of the singular strategy were based on two patches with different habitats linked by migration [26]. In such a case, limiting migration between patches favours specialisation. When considering a more complex system, however, migration between habitats can be altered either by changing dispersal or by changing the spatial distribution of habitats, and in particular their aggregation level. The model developed here predicts that both effects determine the fate of the metapopulation: either remaining monomorphic or evolving into specialists of each habitat. As underlined by Débarre & Gandon [25], spatial and two-patch models reach the same conclusion that limiting migration between habitats favours specialisation. Nevertheless, we were able here to quantify this effect in several other dispersal contexts. When considering a metapopulation that develops in a hierarchical network of patches, we showed that the branching criterion splits into a non-spatial term that depends on the fitness function and on the global proportion of habitats and a spatial term that reflects habitat allocation to groups of neighbouring patches. When considering a lattice network, we showed that for a short dispersal range the spatial distribution of habitats is the most influential factor on branching whereas its effects decrease relative to those of habitat proportions and habitat differentiation when the dispersal range increases.

The evolutionary speed of a monomorphic population is approximately given by the canonical equation of adaptive dynamics. We found that the distance up to the singular strategy decreases exponentially and that it depends on mutation amplitude, mutation rate, habitat selectivity and offspring variability. In addition, limiting dispersal was found to reduce the time to reach the singular strategy. Habitat aggregation did not always influence the evolutionary speed towards the singular strategy and more work is needed to better characterise the role of habitat spatial structure in the evolutionary speed of a monomorphic population. In addition, we used a deterministic model, and thus we did not take into account the impact of random genetic drift. However it is known that finite population size could delay evolutionary branching via demographic stochasticity. For example Claessen *et al*. [47] used an individual based model to study how evolutionary branching is affected by demographic stochasticity. Their key finding is that in small populations branching can be delayed and even avoided. The effect of demographic stochasticity on the trait substitution sequence and the canonical equation was also studied by Champagnat & Lambert [48].

Evolution after branching is little discussed in the literature. Meszéna *et al*. [26] demonstrated that adaptation of coexisting phenotypes to habitats is stronger for higher habitat differentiation or lower migration rates. Using simulations, we showed that the spatial distribution of habitats impacts both the mean phenotype of specialist phenotypic morphs and their phenotypic variance: specialists were found better adapted when habitat aggregation level was high. Moreover, specialists evolved faster towards their optimal phenotype when habitats were more aggregated. In this work we established the singular strategy and the branching criterion expression in the general case but only explored the post branching evolution when the environment was composed of two habitats. Geritz *et al*. [21] studied the stable strategies that coexisted in an environment composed of three habitats. They found that when a population underwent evolutionary branching, it could split into two or three phenotypic morphs depending on habitat differentiation. When two morphs were selected, they had an intermediate phenotype between two of the habitat optima. However the spatial structure of habitats could modify this result. For example, increasing the aggregation level of only one of the three habitats may lead to the selection for its specialist whereas a generalist could be maintained on the two habitats remaining mixed. Neverthless this prediction should be refined by studying more complex environment compositions and structures.

Coexistence of specialist and generalist genotypes in heterogeneous landscapes has been investigated by Débarre & Lenormand [31] who studied the output of competition among pre-defined genotypes. They showed that the three genotypes can coexist in a two-habitat landscape whereas this was never the case with our approach. This underlines an important difference between the simulation of a gradual evolution of a population and that of competition between pre-existing genotypes. In the first case, the population first evolves towards a generalist genotype and then may split into two coexisting specialists. When genotypes pre-exist and are not allowed to evolve through mutation, their competition may lead to the coexistence of three genotypes. To which extent this is possible in the presence of mutations remains an open question.

Adaptive dynamics allows studying the long-term evolution of a population. This framework is appropriate for modelling the evolution of short life cycles organisms such as bacteria or fungi over one or several years. In our study, habitat structure did not change over time. However, the environment is not static and can suffer important seasonal changes. For example, the Australian native plant *Linum marginale* undergoes two reverse patterns in its life-cycle according to climatic regions. It has apparent shoots from spring to autumn and overwinters as underground root-stocks in the sub-alpine regions of New South Wales (Australia) and, in the drier regions, plants grow through winter and flower in early spring with summer survival largely being achieved via protected rootstock. These reverse patterns impose strong spatio-temporal heterogeneity which drives the evolution of the pathogen *Melampsora lini* [49]. In an agricultural landscape crop rotations over time represent drastic seasonal changes which potentially impact the evolution of pathogen populations [50]. Such crop rotations could be used to develop strategies for controlling epidemics in the long term [51]. A perspective of this work could be to study how temporal heterogeneities alter population evolution.

The concept of ecological niche is at the heart of many management strategies of species conservation or pest management [52]. Processes that occur at the landscape scale are increasingly gaining attention and, by showing that spatial structures are crucial in determining the specialisation level and the evolutionary speed of a population, our results give insight on how spatial heterogeneity drives the niche breadth of species. In the particular field of landscape epidemiology [53], still very few studies incorporate large scale processes and most of them deal with controlling epidemic spread [54], [55] without pathogen evolution. Most predictions on the durability of host resistance with regard to pathogen adaptation rely on a few non-spatial models [56]. Our model better takes account of mutation and spatial structures and strongly suggests that landscape spatial organisation impacts pathogen evolution. This potentially opens the way to the design of strategies for a sustainable use of resistant varieties in agriculture.

## Supporting Information

Figure S1 **Illustration of the measures used to characterize the evolutionary trajectories.**
(PDF)

Figure S2 **Comparison between analytical and simulation results.**
(PDF)

Figure S3 **Time to reach the singular strategy.**
(PDF)

Figure S4 **Evolution of the within population phenotypic variance and global evolutionary trajectory.**
(PDF)

Figure S5 **Level of local adaptation.**
(PDF)

Appendix S1 **Invasion analysis.**
(PDF)

Appendix S2 **Canonical equation of adaptive dynamics.**
(PDF)

Appendix S3 **Hierarchical metapopulation.**
(PDF)

Appendix S4 **Definition of measures on simulated evolution.**
(PDF)

Appendix S5 **Comparing theoretical predictions and simulation results.**
(PDF)
