# Supplementary material for: Dynamics of Adaptation in Spatially Heterogeneous Metapopulations
Source: PLoS One. 2013 Feb 12;8(2):e54697. doi: 10.1371/journal.pone.0054697 (PMC3570538; doi:10.1371/journal.pone.0054697)
Supplement: Figure S1 — Illustration of the measures used to characterize the evolutionary trajectories. (PDF) [file pone.0054697.s001.pdf]

Supporting information: Dynamics of adaptation in spatially heterogeneous metapopulations

J. Papaïx [1,2,a], O. David [2,b], C. Lannou [1,c] & H. Monod [2,d]

26/12/2012

[1]INRA, UMR 1290 BIOGER, F-78850 Thiverval Grignon.

[2]INRA, UR 341 Mathématiques et Informatique Appliquées, F-78350 Jouy-en-Josas.

[a]julien.papaix@jouy.inra.fr

[b]olivier.david@jouy.inra.fr

[c]christian.lannou@grignon.inra.fr

[d]herve.monod@jouy.inra.fr

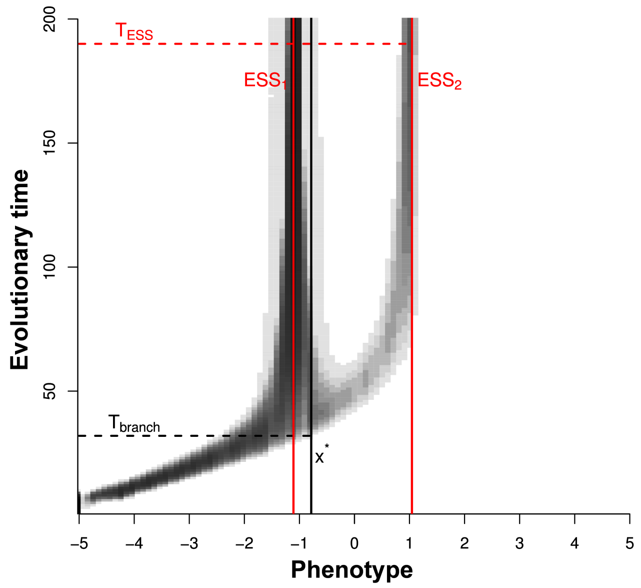

Figure S1. Illustration of the measures used to characterize the evolutionary trajectories (see Appendix S4 for more details). $\hat{x}^{\star}$: estimated singular strategy; T_{branch}: time to reach the singular strategy; ESS_1 and ESS_2: trait values of the specialists; T_{ESS}: time to reach the specialists. Parameters are $m_s = 15\%$, $\delta/\sigma = 1.1$ ($\delta = -\beta_1 = \beta_2 = 1.1$ and $\sigma = 1$), $\pi = 0.16$ and AI $= 0.7$ (situation B). The lattice environment was used.
