# Supplementary material for: Dynamics of Adaptation in Spatially Heterogeneous Metapopulations
Source: PLoS One. 2013 Feb 12;8(2):e54697. doi: 10.1371/journal.pone.0054697 (PMC3570538; doi:10.1371/journal.pone.0054697)
Supplement: Figure S3 — Time to reach the singular strategy. (PDF) [file pone.0054697.s003.pdf]

Supporting information: Dynamics of adaptation in spatially heterogeneous metapopulations

J. Papaïx [1,2,a], O. David [2,b], C. Lannou [1,c] & H. Monod [2,d]

26/12/2012

[1]INRA, UMR 1290 BIOGER, F-78850 Thiverval Grignon.

[2]INRA, UR 341 Mathématiques et Informatique Appliquées, F-78350 Jouy-en-Josas.

[a]julien.papaix@jouy.inra.fr

[b]olivier.david@jouy.inra.fr

[c]christian.lannou@grignon.inra.fr

[d]herve.monod@jouy.inra.fr

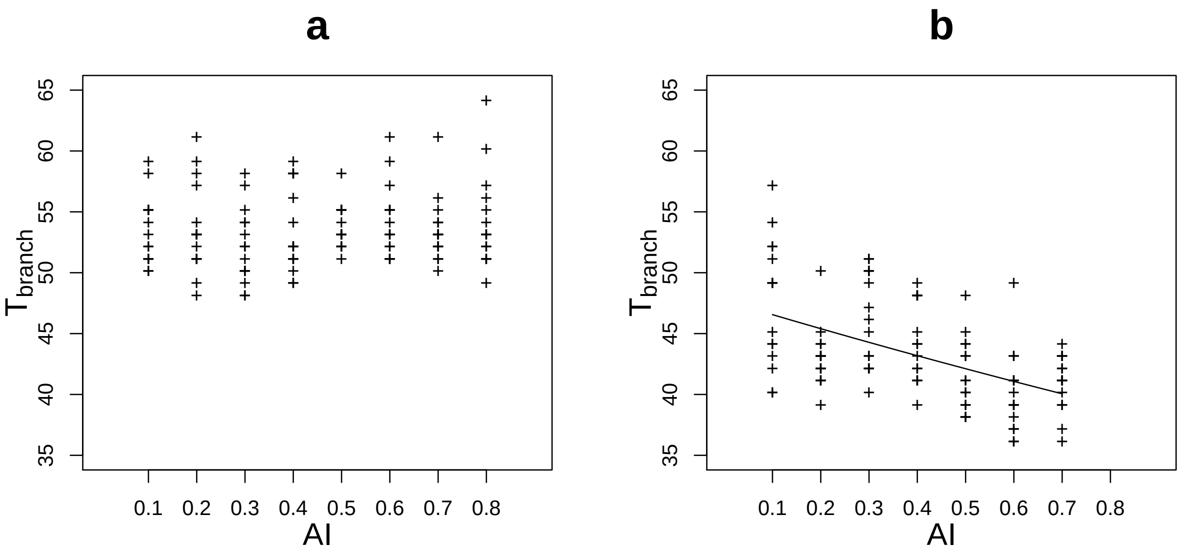

Figure S3. The time to reach the singular strategy (T_{branch}) plotted against the aggregation level of habitat 2 (AI) in the case of situation A (a) and situation B (b). The relationship between T_{branch} and AI was tested by a GLM with Poisson distributed errors. The solid line indicates that the slope was significantly different from 0 with a 0.001 threshold. Other parameters are: (a) $m_s = 0.75$, $\delta/\sigma = 1.1$ ($\delta = -\beta_1 = \beta_2 = 1.1$ and $\sigma = 1$) and $\pi = 0.32$; (b) $m_s = 0.15$, $\delta/\sigma = 1.1$ ($\delta = -\beta_1 = \beta_2 = 1.1$ and $\sigma = 1$) and $\pi = 0.16$. The lattice environment was used.
