# Supplementary material for: Dynamics of Adaptation in Spatially Heterogeneous Metapopulations
Source: PLoS One. 2013 Feb 12;8(2):e54697. doi: 10.1371/journal.pone.0054697 (PMC3570538; doi:10.1371/journal.pone.0054697)
Supplement: Figure S4 — Evolution of the within population phenotypic variance and global evolutionary trajectory. (PDF) [file pone.0054697.s004.pdf]

Supporting information: Dynamics of adaptation in spatially heterogeneous metapopulations

J. Papaïx [1,2,a], O. David [2,b], C. Lannou [1,c] & H. Monod [2,d]

26/12/2012

[1]INRA, UMR 1290 BIOGER, F-78850 Thiverval Grignon.

[2]INRA, UR 341 Mathématiques et Informatique Appliquées, F-78350 Jouy-en-Josas.

[a]julien.papaix@jouy.inra.fr

[b]olivier.david@jouy.inra.fr

[c]christian.lannou@grignon.inra.fr

[d]herve.monod@jouy.inra.fr

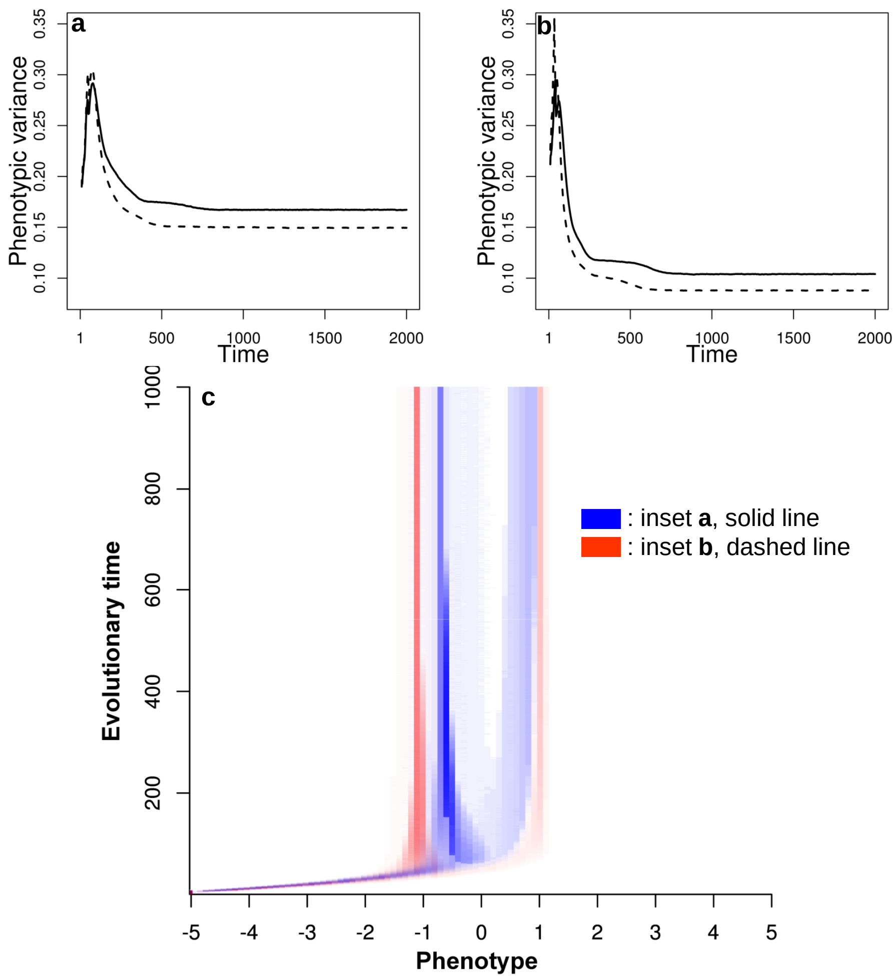

Figure S4. Evolution of the within population phenotypic variance (**a** and **b**) and global evolutionary trajectory (**c**). Solid line: AI = 0.1; dashed line: AI = 0.7. Other parameters are: situation A (**a**), $m_s = 75\%$, $\delta/\sigma = 1.1$ ($\delta = -\beta_1 = \beta_2 = 1.1$ and $\sigma = 1$) and $\pi = 0.32$; situation B (**b**), $m_s = 15\%$, $\delta/\sigma = 1.1$ ($\delta = -\beta_1 = \beta_2 = 1.1$ and $\sigma = 1$) and $\pi = 0.16$. The lattice environment was used.
