# Supplementary material for: Dynamics of Adaptation in Spatially Heterogeneous Metapopulations
Source: PLoS One. 2013 Feb 12;8(2):e54697. doi: 10.1371/journal.pone.0054697 (PMC3570538; doi:10.1371/journal.pone.0054697)
Supplement: Figure S5 — Level of local adaptation. (PDF) [file pone.0054697.s005.pdf]

Supporting information: Dynamics of adaptation in spatially heterogeneous metapopulations

J. Papaïx [1,2,a], O. David [2,b], C. Lannou [1,c] & H. Monod [2,d]

26/12/2012

[1]INRA, UMR 1290 BIOGER, F-78850 Thiverval Grignon.

[2]INRA, UR 341 Mathématiques et Informatique Appliquées, F-78350 Jouy-en-Josas.

[a]julien.papaix@jouy.inra.fr

[b]olivier.david@jouy.inra.fr

[c]christian.lannou@grignon.inra.fr

[d]herve.monod@jouy.inra.fr

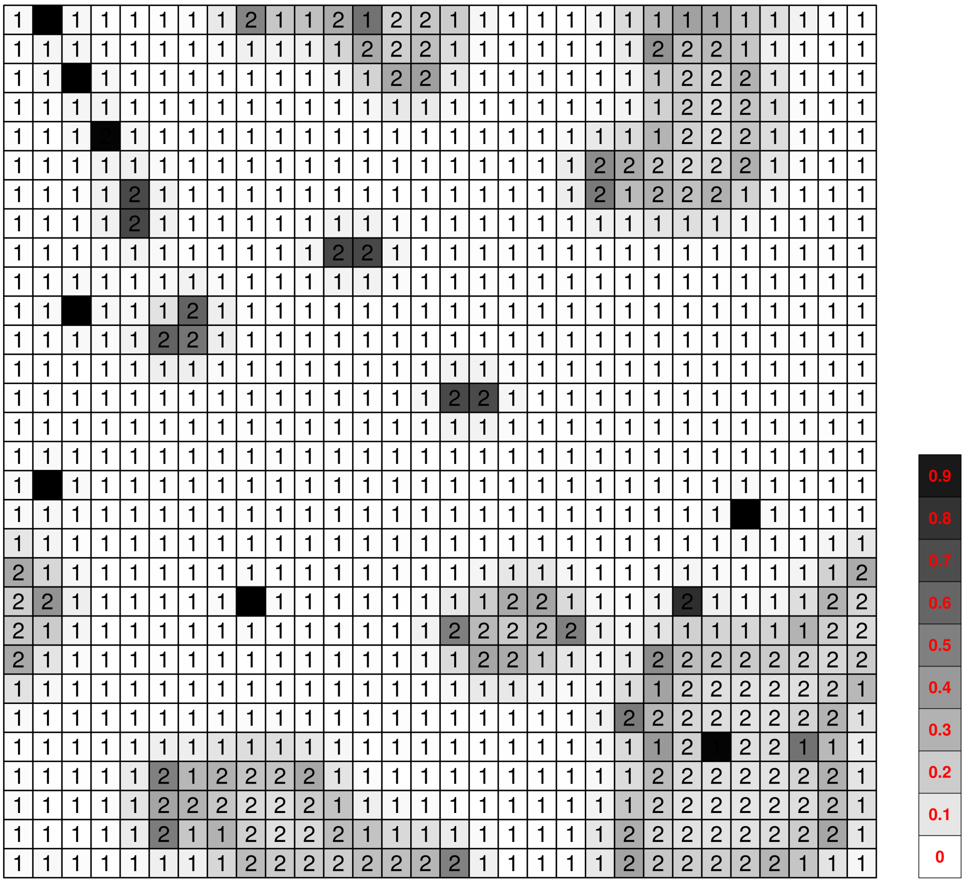

Figure S5. Level of local adaptation at the end of a simulation run. The grey scale indicates the mismatch between the mean phenotype ($\bar{x}_j(t)$) and the optimal phenotype ($\beta_{h(j)}$): $|\bar{x}_j(t) - \beta_{h(j)}|$. The more adapted the local population, the lighter the grey. Patches are marked by their habitat 1 or 2. In this case, specialisation was predicted according to the analytical branching criterion. Parameters are $m_s = 15\%$, $\delta/\sigma = 0.8$ ($\delta = -\beta_1 = \beta_2 = 0.8$ and $\sigma = 1$), $\pi = 0.16$ and AI $= 0.7$. The lattice environment was used.
