# Supplementary material for: Dynamics of Adaptation in Spatially Heterogeneous Metapopulations
Source: PLoS One. 2013 Feb 12;8(2):e54697. doi: 10.1371/journal.pone.0054697 (PMC3570538; doi:10.1371/journal.pone.0054697)
Supplement: Appendix S1 — Invasion analysis. (PDF) [file pone.0054697.s006.pdf]

# Supporting information: Dynamics of adaptation in spatially heterogeneous metapopulations

J. Papaïx [1,2,a], O. David [2,b], C. Lannou [1,c] & H. Monod [2,d]

26/12/2012

[1]INRA, UMR 1290 BIOGER, F-78850 Thiverval Grignon.

[2]INRA, UR 341 Mathématiques et Informatique Appliquées, F-78350 Jouy-en-Josas.

[a]julien.papaix@jouy.inra.fr

[b]olivier.david@jouy.inra.fr

[c]christian.lannou@grignon.inra.fr

[d]herve.monod@jouy.inra.fr

# Appendix S1: invasion analysis

## Competition between a mutant and a resident

**Elementary dynamics** The resident population is assumed to be monomorphic with trait value $x_1$. At a given time $t_1$, an individual produces a mutant with trait value $x_2$. At a time $t_2$ shortly after $t_1$ ($t_2 > t_1$, with $t_2 - t_1$ small), the mutant is rare enough to consider that the resident population remains monomorphic ($n_{2j}(t) << n_{1j}(t)$ for all patches $j$ and for $t_1 \leq t \leq t_2$). Under this approximation and according to equation (1) of the main text, the number of mutants in patch $j$ at time $t+1$ is equal to

$$n_{2j}(t+1) \approx \frac{\left(\sum_{j'=1}^{P} m_{j'j}\, n_{2j'}(t)\right) f_{h(j)}(x_2)}{\left(\sum_{j'=1}^{P} m_{j'j}\, K_{j'}\right) f_{h(j)}(x_1)} K_j.$$

Let $N_i(t) = \Big(n_{i1}(t), \cdots, n_{iP}(t)\Big)'$ denote the vector of the population sizes $n_{ij}(t)$ of phenotype $i$ on patches $j = 1, \cdots, P$. The vector of mutant population sizes satisfies the matrix equation $N_2(t+1) = A(x_1, x_2)\, N_2(t)$, where $A(x_1, x_2)$ is the $P \times P$ projection matrix with element in row $j$ and column $j'$ equal to

$$\Big[A(x_1, x_2)\Big]_{jj'} = \frac{m_{j'j}\, \overline{K}_j}{m_{+j}} \frac{f_{h(j)}(x_2)}{f_{h(j)}(x_1)}. \tag{1}$$

Provided that $A(x_1, x_2)$ is irreducible and primitive, the growth of phenotype $x_2$ in the period $t_2$ shortly after $t_1$ is approximately given by

$$N_2(t_2) \approx \big(\lambda^{(1)}(x_1, x_2)\big)^{t_2 - t_1}\, r^{(1)}(x_1, x_2)\, l^{(1)}(x_1, x_2)'\, N_2(t_1), \tag{2}$$

where $\lambda^{(1)}(x_1, x_2)$ is the dominant eigenvalue of $A(x_1, x_2)$, and $l^{(1)}(x_1, x_2)$ and $r^{(1)}(x_1, x_2)$ are its associated left and right normalized eigenvectors, which have all their entries strictly positive (Caswell, 2001, chapter 4). Note that $A(x_1, x_2)$ is irreducible if and only if there is a dispersal path from each patch to every other patch, *i.e.* there are no disconnected subsets of patches or traps in the landscape, with respect to dispersal (Caswell, 2001, chapter 4). It is irreducible and primitive if and only if there exists an integer $q$ such that $A(x_1, x_2)^q$ has all entries strictly positive.

**Mutant's fate** The mutant's fate is determined by its invasion fitness function $s(\cdot, \cdot)$ defined by $s(x_1, x_2) = \ln\big(\lambda^{(1)}(x_1, x_2)\big)$ (Durinx et al., 2008, see also equation (2)). If $s(x_1, x_2) \leq 0$, the mutant becomes extinct shortly after $t_1$. If $s(x_1, x_2) > 0$, then the mutant either becomes extinct shortly after $t_1$ by genetic drift, or it increases in frequency in the population since $l^{(1)}(x_1, x_2)'\, N_2(t_1) > 0$. Because our model is deterministic, only the latter alternative occurs and it occurs in all patches since all coordinates of $r^{(1)}(x_1, x_2)$ are strictly positive.

Mutation steps are assumed to be small. Thus the mutant and resident phenotypes have close trait values $x_1$ and $x_2$. In this case and away from some particular trait values called singular strategies (see below), a stable dimorphism of $x_1$ and $x_2$ is not possible (Champagnat et al., 2006; Durinx et al., 2008). A mutant with positive invasion fitness will eventually replace the resident and will generate a new monomorphic resident population with trait value $x_2$. It is assumed that mutations occur sufficiently infrequently so that the population becomes monomorphic before a new mutant appears.

**Long-term population evolution towards the singular strategy** At a longer time scale, the resident population evolves gradually through a succession of mutant invasions. As mutation steps are small, the first order approximation of the invasion fitness $s(x_1, x_2)$ gives

$$s(x_1, x_2) \approx s(x_1, x_1) + (x_2 - x_1).D_{\text{loc}}(x_1),$$

where $D_{\text{loc}}(x_1) = \dfrac{\partial s}{\partial x_2}(x_1, x_1)$ is the local fitness gradient (Geritz et al., 1998). Its sign determines the direction of selection: if $D_{\text{loc}}(x_1) > 0$, then only mutants with $x_2 > x_1$ can invade, whereas if $D_{\text{loc}}(x_1) < 0$, then this is possible for mutants with $x_2 < x_1$ only. The trait value $x^\star$ for which the local fitness gradient is zero, *i.e.* $D_{\text{loc}}(x^\star) = 0$, is called the 'singular strategy'. It is said to be convergence stable if the resident population evolves towards $x^\star$, which occurs if the derivative of the local fitness gradient $D_{\text{loc}}(x)$ is negative at $x = x^\star$ (Geritz et al., 1998). If $D_{\text{loc}}(x^\star) > 0$, then $x^\star$ is an evolutionary repeller.

**Notations and conventions** In the following, explicit reference to $(x_1, x_2)$ will often be omitted from quantities such as the matrix $A(x_1, x_2)$ or its eigenvalues and eigenvectors. This is in order to simplify notations, but also because we may view these quantities as functions and the relationships between them as functional relationships. For example equations involving $l^{(j)}(x_1, x_2)$ and $r^{(j)}(x_1, x_2)$ can be considered as functional equations between the functions $l^{(j)}(\cdot,\cdot)$ and $r^{(j)}(\cdot,\cdot)$ of $(x_1, x_2)$. Besides, the derivative of any given function $g(x_1, x_2)$ with respect to $x_1$ or $x_2$ will be denoted by $g_{x_1}$ or $g_{x_2}$ respectively. For example, $\frac{\partial s}{\partial x_2}(x_1, x_2)$ will be denoted by $s_{x_2}(x_1, x_2)$ or, more concisely, by $s_{x_2}$.

The eigenvectors of matrix $A$ are scaled so that $l^{(j)\prime} r^{(j)} = 1$ and $l^{(j)\prime} r^{(j')} = 0$ for $j \neq j'$. The dominant right eigenvector $r^{(1)}$ is scaled to satisfy $\sum_j [r^{(1)}]_j = 1$. For simplicity all the eigenvalues $\lambda^{(j)}$ and the eigenvectors $l^{(j)}$ and $r^{(j)}$ of $A$ are assumed to be real in this appendix.

**Properties of $A(x, x)$** The matrix $A(x, x)$ is independent of $x$ since $\big[A(x, x)\big]_{jj'} = \dfrac{m_{j'j}\overline{K}_j}{m_{+j}}$. We shall denote it by $A_{\text{env}}$ since it depends only on the environment structure. It follows that the eigenvalues, left eigenvectors and right eigenvectors of $A_{\text{env}}$ are independent of $x$ too. They will be denoted by $\lambda^{(j)}_{\text{env}}$, $l^{(j)}_{\text{env}}$, $r^{(j)}_{\text{env}}$ respectively. In the absence of a mutant, the metapopulation is at equilibrium and the dominant eigenvalue of $A_{\text{env}}$ satisfies $\lambda^{(1)}_{\text{env}} = 1$. The dominant right eigenvector satisfies $\big[r^{(1)}_{\text{env}}\big]_j = \overline{K}_j$ and $A_{\text{env}}\, r^{(1)}_{\text{env}} = r^{(1)}_{\text{env}}$.

**Properties of the derivative of $A$ and its eigenvalues** We remind that all the eigenvectors are scaled to satisfy $l^{(j)\prime}r^{(j)} = 1$. In that case, the eigenvalue derivatives satisfy (Caswell, 2001, Chapter 9, eqn. 9.10)

$$\lambda_{x_2}^{(j)} = l^{(j)\prime}\, A_{x_2}\, r^{(j)}. \tag{3}$$

By deriving equation (1), we obtain

$$[A_{x_2}(x_1, x_2)]_{jj'} = \frac{m_{j'j}\overline{K}_j}{m_{+j}} \cdot \frac{f_{h(j)}(x_2)}{f_{h(j)}(x_1)} \cdot \frac{\beta_{h(j)} - x_2}{\sigma^2}$$

which gives the following two properties on the derivative of $A$ with respect to $x_2$ :

$$A_{x_2} = \frac{1}{\sigma}\,\Delta\, A, \tag{4}$$

$$A_{x_2}\, r^{(j)} = \frac{1}{\sigma}\,\lambda^{(j)}\,\Delta\, r^{(j)}, \tag{5}$$

where $\Delta$ is the $P \times P$ diagonal matrix depending on $x_2$, whose $j$th diagonal element equals $(\beta_{h(j)} - x_2)/\sigma$.

## Singular strategy

In this section, we determine an analytical expression of the singular strategy $x^\star$.

**Mathematical expression of the singular strategy** We look for the singular value $x^\star$ that satisfies $D_{\text{loc}}(x^\star) = 0$. First, we calculate the fitness gradient $s_{x_2}(x_1, x_2)$. Using equations (3) and (5), we get

$$\begin{aligned} s_{x_2} &= \ln\left(\lambda^{(1)}\right)_{x_2} \\ &= \frac{1}{\lambda^{(1)}} \cdot \lambda^{(1)}_{x_2} \\ &= \frac{1}{\lambda^{(1)}} \cdot l^{(1)\prime}\, A_{x_2}\, r^{(1)} \\ &= \frac{1}{\sigma} \cdot l^{(1)\prime}\,\Delta\, r^{(1)}. \end{aligned}$$

Second we deduce the following expression of the local fitness gradient $D_{\text{loc}}(x) = s_{x_2}(x,x)$ :

$$
\begin{aligned}
D_{\text{loc}}(x) &= \frac{1}{\sigma^2}\sum_j \left( \left[l_{\text{env}}^{(1)}\right]_j \left(\beta_{h(j)} - x\right) \overline{K}_j \right) \\
&= \frac{1}{\sigma^2}\left(x^\star - x\right) \qquad (6)
\end{aligned}
$$

where $x^\star$ is defined by

$$
x^\star = \sum_j \left[l_{\text{env}}^{(1)}\right]_j \overline{K}_j \, \beta_{h(j)} = \sum_k \left( \sum_{j,h(j)=k} \left[l_{\text{env}}^{(1)}\right]_j \overline{K}_j \right) \beta_k .
$$

A strategy is singular iff the local fitness gradient $D_{\text{loc}}(x)$ is zero. Thus $x^\star$ is the singular strategy. From equation (6), $\frac{dD_{\text{loc}}}{dx}(x) = -\frac{1}{\sigma^2} < 0$, so that the singular strategy $x^\star$ is always convergence stable.

## Stability of the singular strategy and branching criterion

In this section, we study the fitness gradient and deduce the branching condition given in Section 3.1.2 of the main text.

Once the resident population has reached the convergence-stable singular strategy $x^\star$, it may remain monomorphic with phenotype $x^\star$ or branch into distinct phenotypes better adapted to the different habitats. In the first case, the singular strategy is said to be evolutionarily stable. The second order approximation of the invasion fitness $s(x_1, x_2)$ gives

$$
s(x_1,x_2) \approx s(x_1,x_1) + (x_2 - x_1).D_{\text{loc}}(x_1) + \frac{(x_2-x_1)^2}{2} \, . \, D_{\text{loc}}^{(2)}(x_1), \qquad (7)
$$

with $D_{\text{loc}}^{(2)}(x_1) = \frac{\partial^2 s}{\partial x_2^2}(x_1,x_1) = s_{x_2x_2}(x_1,x_1)$. The stability of $x^\star$ is determined by the third term of equation (7). If $D_{\text{loc}}^{(2)}(x^\star) > 0$, then $x^\star$ is a branching point. On the contrary, if $D_{\text{loc}}^{(2)}(x^\star) < 0$, then $x^\star$ is an 'evolutionarily stable strategy' (Geritz et al., 1998).

In order to know if the strategy $x^\star$ is evolutionarily stable, we calculate $D_{\text{loc}}^{(2)}(x^\star)$. We have :

$$
\begin{aligned}
s_{x_2x_2} &= \ln\left(\lambda^{(1)}\right)_{x_2x_2} \\
&= \frac{1}{\lambda^{(1)}}\lambda^{(1)}_{x_2x_2} - \left(\frac{1}{\lambda^{(1)}}\lambda^{(1)}_{x_2}\right)^2 \\
&= \frac{1}{\lambda^{(1)}}\lambda^{(1)}_{x_2x_2} - s_{x_2}^2 .
\end{aligned}
$$

Since $\lambda^{(1)}(x^\star, x^\star) = \lambda^{(1)}_{\text{env}} = 1$ and $s_{x_2}(x^\star, x^\star) = D_{\text{loc}}(x^\star) = 0$, we get

$$D^{(2)}_{\text{loc}}(x^\star) = \lambda^{(1)}_{x_2x_2}(x^\star, x^\star).$$

By deriving equation (3) with $j = 1$ we have:

$$\lambda^{(1)}_{x_2x_2} = l^{(1)\prime}_{x_2} A_{x_2} r^{(1)} + l^{(1)\prime} A_{x_2x_2} r^{(1)} + l^{(1)\prime} A_{x_2} r^{(1)}_{x_2}.$$

This is the sum of three terms which we have to evaluate at $x_1 = x_2 = x^\star$ to get $D^{(2)}_{\text{loc}}(x^\star)$. We denote these three terms evaluated at $x_1 = x_2 = x^\star$ by $\omega_1^\star$, $\omega_2^\star$ and $\omega_3^\star$.

**First term computation** According to Caswell (2001, chapter 9, eqn. 9.132) and using equation (5),

$$\begin{aligned} l^{(1)}_{x_2} &= \sum_{j,j\neq 1} \frac{l^{(1)\prime} A_{x_2} r^{(j)}}{\lambda^{(1)} - \lambda^{(j)}} l^{(j)} \\ &= \frac{1}{\sigma} \sum_{j,j\neq 1} \frac{\lambda^{(j)}}{\lambda^{(1)} - \lambda^{(j)}} l^{(1)\prime} \Delta r^{(j)} l^{(j)}. \end{aligned}$$

By equation (5) again

$$l^{(1)\prime}_{x_2} A_{x_2} r^{(1)} = \frac{1}{\sigma} \lambda^{(1)} l^{(1)\prime}_{x_2} \Delta r^{(1)}.$$

When evaluated at $x_1 = x_2 = x^\star$, this expression gives the first term of $D^{(2)}_{\text{loc}}(x^\star)$, that is,

$$\omega_1^\star = \frac{1}{\sigma^2} \lambda^{(1)}_{\text{env}} l^{(1)\prime}_{\text{env}} \Delta^\star \left( \sum_{j=2}^{P} \frac{\lambda^{(j)}_{\text{env}}}{\lambda^{(1)}_{\text{env}} - \lambda^{(j)}_{\text{env}}} r^{(j)}_{\text{env}} l^{(j)\prime}_{\text{env}} \right) \Delta^\star r^{(1)}_{\text{env}},$$

where $\Delta^\star = \Delta(x^\star)$.

**Second term computation** Let $I_P$ denote the identity matrix of order $P$. By deriving equation (4) we have:

$$\begin{aligned} A_{x_2x_2} &= \frac{1}{\sigma} \Delta A_{x_2} + \frac{1}{\sigma} \Delta_{x_2} A \\ &= \frac{1}{\sigma^2} \left( \Delta^2 - I_P \right) A. \end{aligned}$$

It follows that,

$$\begin{aligned} l^{(1)\prime} A_{x_2 x_2} r^{(1)} &= \frac{1}{\sigma^2}\, l^{(1)\prime} \left(\Delta^2 - I_P\right) A\, r^{(1)} \\ &= \frac{1}{\sigma^2}\, \lambda^{(1)} \, \left(l^{(1)\prime}\Delta^2 r^{(1)} - 1\right). \end{aligned}$$

When evaluated at $x_1 = x_2 = x^\star$, it gives the second term of $D^{(2)}_{\text{loc}}(x^\star)$ :

$$\omega_2^\star = \frac{1}{\sigma^2}\, \lambda^{(1)}_{\text{env}} \, \left(l^{(1)}_{\text{env}}{}'\Delta^{\star 2} r^{(1)}_{\text{env}} - 1\right).$$

**Third term computation** According to Caswell (2001, chapter 9, eqn. 9.131),

$$\begin{aligned} r^{(1)}_{x_2} &= \sum_{j=2}^{P} \frac{l^{(j)\prime} A_{x_2}\, r^{(1)}}{\lambda^{(1)} - \lambda^{(j)}}\, r^{(j)} \\ &= \frac{1}{\sigma}\lambda^{(1)} \sum_{j=2}^{P} \frac{l^{(j)\prime}\Delta r^{(1)}}{\lambda^{(1)} - \lambda^{(j)}}\, r^{(j)}. \end{aligned}$$

By equation (4)

$$l^{(1)\prime} A_{x_2}\, r^{(1)}_{x_2} = \frac{1}{\sigma}\, l^{(1)\prime} \Delta A\, r^{(1)}_{x_2}.$$

When developed and evaluated at $x_1 = x_2 = x^\star$, this expression gives the third term of $D^{(2)}_{\text{loc}}(x^\star)$, which is equal to the first one : $\omega_3^\star = \omega_1^\star$.

**Global formula** Finally, since $\lambda^{(1)}_{\text{env}} = 1$ we have,

$$\begin{aligned} D^{(2)}_{\text{loc}}(x^\star) &= \omega_1^\star + \omega_2^\star + \omega_3^\star \\ &= \frac{1}{\sigma^2}\left[ l^{(1)}_{\text{env}}{}'\, \Delta^\star \left( I_P + 2\sum_{j=2}^{P} \frac{\lambda^{(j)}_{\text{env}}}{1-\lambda^{(j)}_{\text{env}}}\, r^{(j)}_{\text{env}} l^{(j)}_{\text{env}}{}' \right) \Delta^\star\, r^{(1)}_{\text{env}} - 1 \right]. \end{aligned}$$

The strategy $x^\star$ is evolutionarily stable if

$$l^{(1)}_{\text{env}}{}'\, \Delta^\star \left( I_P + 2\sum_{j=2}^{P} \frac{\lambda^{(j)}_{\text{env}}}{1-\lambda^{(j)}_{\text{env}}}\, r^{(j)}_{\text{env}} l^{(j)}_{\text{env}}{}' \right) \Delta^\star\, r^{(1)}_{\text{env}} - 1 < 0$$

which is equivalent to the condition given in Section 3.1.2 of the main text.

# References

H Caswell. *Matrix Population Models.* Sinauer Associates Inc. Publishers, Sunderland (USA), 2001.

N Champagnat, R Ferrière, and S Méléard. Unifying evolutionary dynamics: From individual stochastic processes to macroscopic models. *Theoretical Population Biology*, 69:297–321, 2006.

M Durinx, J A J H Metz, and G Meszéna. Adaptive dynamics for physiologically structured population models. *Journal of Mathematical Biology*, 56:673–742, 2008.

S A H Geritz, E Kisdi, G Meszéna, and J A J H Metz. Evolutionarily singular strategies and the adaptive growth and branching of the evolutionary tree. *Evolutionary Ecology*, 12:35–57, 1998.
