# Supplementary material for: Dynamics of Adaptation in Spatially Heterogeneous Metapopulations
Source: PLoS One. 2013 Feb 12;8(2):e54697. doi: 10.1371/journal.pone.0054697 (PMC3570538; doi:10.1371/journal.pone.0054697)
Supplement: Appendix S2 — Canonical equation of adaptive dynamics. (PDF) [file pone.0054697.s007.pdf]

Supporting information: Dynamics of adaptation in spatially heterogeneous metapopulations

J. Papaïx [1,2,a], O. David [2,b], C. Lannou [1,c] & H. Monod [2,d]

26/12/2012

[1]INRA, UMR 1290 BIOGER, F-78850 Thiverval Grignon.

[2]INRA, UR 341 Mathématiques et Informatique Appliquées, F-78350 Jouy-en-Josas.

[a]julien.papaix@jouy.inra.fr

[b]olivier.david@jouy.inra.fr

[c]christian.lannou@grignon.inra.fr

[d]herve.monod@jouy.inra.fr

Appendix S2: canonical equation of adaptive dynamics

General case

The canonical equation of adaptive dynamics (Champagnat et al., 2006; Durinx et al., 2008) gives the speed of the evolution process towards the singular strategy. We recall its origin and give its expression in our setting. We consider the evolution of the quantitative trait x in a monomorphic population before x reaches the singular strategy $x^{\star}$. The process is now considered as stochastic and its asymptotic behaviour is studied. The population size K_T is assumed to be large.

At the finest time scale, mutations are assumed to be rare and the probability of a mutant birth in the population per generation is assumed to be equal to a very small value $\theta_0(x)$. At a larger timescale, time is continuous and one unit lasts n_G generations of the finest time scale, where n_G tends to infinity. In this larger timescale, mutations approximately occur according to a continuous-time Poisson process with rate $n_G\,\theta_0(x)$. Asymptotic results are obtained by assuming

that n_G and K_T tend to infinity at the same rate and θ_0 tends to zero, while $\theta(x) = \lim_{n_G \to +\infty} n_G \, \theta_0(x)$ is of order one.

Mutations are assumed to be small. More precisely, the trait of a mutant is given by $x_2 = x + \varepsilon z$ where ε is a small scalar and the random variable z is assumed to have a centred and symmetrical mutation distribution μ_x. The timescale is changed a second time so that one unit in the new timescale lasts ε^{-2} units in the previous timescale, where ε tends to zero. Then the trait of the resident follows the equation (Champagnat et al., 2006; Durinx et al., 2008):

$$\dot{x} = \frac{1}{2} \gamma^2(x) \, \theta(x) \, p_{x_2}(x, x)$$

where $\dot{x} = dx/dt$, $\gamma^2(x)$ is the variance of the mutation distribution μ_x, $p(x_1, x_2)$ is the mutant's survival probability and $p_{x_2}(x, x)$ is its partial derivative with respect to x_2 evaluated at $x_1 = x_2 = x$.

Now we calculate the survival probability $p(x_1, x_2)$. If $s(x_1, x_2) < 0$, the mutant cannot survive and $p(x_1, x_2) = 0$. If $s(x_1, x_2) > 0$, the extinction of the mutant population mainly occurs at the beginning of the population growth when the population is still small. Thus, the demography of the mutant population is modelled with a slightly supercritical multi-type branching process with a reproduction mean matrix equal to $A(x_1, x_2)$ (Durinx et al., 2008). Let $\xi_{j'j}(x_1)$ denote the random number of descendants in patch j of an individual with trait x_1 in patch j' in a resident population with trait x_1. Then, provided the eigenvectors are scaled so that $l_{\text{env}}^{(1)}{}' r_{\text{env}}^{(1)} = 1$ and $\sum_j [r_{\text{env}}^{(1)}]_j = 1$ (as stated before), the survival probability of a mutant that arises in patch j is equal to (Durinx et al., 2008; Haccou et al., 2005, section 5.6):

$$p_j(x_1, x_2) \approx 2s(x_1, x_2)[l_{\text{env}}^{(1)}]_j / \tau^2(x_1),$$

where

$$\tau^2(x) = \sum_{j'} [r_{\text{env}}^{(1)}]_{j'} \, \text{Var}\Big[\sum_j [l_{\text{env}}^{(1)}]_j \, \xi_{j'j}(x) \Big]. \tag{1}$$

The mutant arises in patch j with probability $\left[r_{\text{env}}^{(1)}\right]_j = \overline{K}_j$. Thus the global survival probability of the mutant is equal to:

$$p(x_1, x_2) = \sum_j p_j(x_1, x_2)[r_{\text{env}}^{(1)}]_j \approx 2s(x_1, x_2)/\tau^2(x_1),$$

since $l_{\text{env}}^{(1)\prime} r_{\text{env}}^{(1)} = 1$. Deriving this equation with respect to x_2, we obtain:

$$p_{x_2}(x, x) \approx 2 s_{x_2}(x, x)/\tau^2(x).$$

Applying equation (6) of Appendix S1, it follows that the canonical equation is equal to:

$$\dot{x} = \frac{\gamma^2(x)\,\theta(x)}{\tau^2(x)\,\sigma^2}(x^\star - x).$$

Only $\tau^2(x)$ can depend on the landscape structure through $\xi_{j'j}(x)$, $l_{\text{env}}^{(1)}$ and $r_{\text{env}}^{(1)}$.

Symmetric case

A simple way of introducing demographic stochasticity in the model is to assume that each patch has a large offspring pool from which the next generation is sampled. The offspring of a particular resident individual in patch j' is assumed to be in proportion $\dfrac{m_{j'j}}{m_{+j} K_T}$ (where $m_{+j} = \sum_{j'} m_{j'j} \overline{K}_{j'}$) in the pool of patch j, from which K_j individuals are sampled. The numbers of descendants $\xi_{j'1}, \ldots, \xi_{j'P}$ of this individual in the different patches are then assumed to be independent with:

$$\xi_{j'j} \sim \mathcal{B}\Big(K_j, \frac{m_{j'j}}{m_{+j} K_T}\Big),$$

where $\mathcal{B}$ denotes the binomial distribution. The expectation of this distribution is consistent with the deterministic model of equation (1) in Appendix S1.

Consider the case of a symmetric environment in the sense that, for all patches j and j', $K_j = K_T/P$ (same carrying capacity K), $m_{+j} = 1/P$ (same input connection) and $m_{j'j} = m_{jj'}$ (symmetric dispersal). Note that the hierarchical environment as defined in the main text is a special case. Then we have $\xi_{j'j} \sim \mathcal{B}\Big(K, \dfrac{m_{j'j}}{K}\Big)$. Besides, the dominant eigenvectors satisfy

$l_{\text{env}}^{(1)} = 1_P$ and $r_{\text{env}}^{(1)} = 1_P/P$. Consequently equation (1) gives:

$$
\begin{aligned}
\tau^2 &= \sum_{j'} [r_{\text{env}}^{(1)}]_{j'} \operatorname{Var}\Big[\sum_j [l_{\text{env}}^{(1)}]_j \, \xi_{j'j}\Big] \\
&= \frac{1}{P} \sum_{j'} \sum_j \operatorname{Var}(\xi_{j'j}) \\
&= \frac{1}{P} \sum_{j'} \sum_j K \frac{m_{j'j}}{K} \Big(1 - \frac{m_{j'j}}{K}\Big) \\
&= \frac{1}{P} \sum_{j'} \Big(\sum_j m_{j'j} - \frac{1}{K} \sum_j m_{j'j}^2\Big) \\
&= \frac{1}{P} \sum_{j'} \Big(1 - \frac{1}{K} \sum_j m_{j'j}^2\Big) \\
&= 1 - \frac{1}{PK} \sum_{j'} \sum_j m_{j'j}^2.
\end{aligned}
$$

Let $\operatorname{Var}_M = \big(\sum_{j',j} m_{j'j}^2 - 1\big)/P^2$ denote the variance of the dispersal rates $m_{j'j}$. We get

$$
\tau^2 = \frac{K_T - 1}{K_T} - \frac{P}{K} \operatorname{Var}_M.
$$

References

N Champagnat, R Ferrière, and S Méléard. Unifying evolutionary dynamics: From individual stochastic processes to macroscopic models. *Theoretical Population Biology*, 69:297–321, 2006.

M Durinx, J A J H Metz, and G Meszéna. Adaptive dynamics for physiologically structured population models. *Journal of Mathematical Biology*, 56:673–742, 2008.

P Haccou, P Jagers, and VA Vatutin. *Branching Processes: Variation, Growth, and Extinction of Populations*. Cambridge University Press, Cambridge (GB), 2005.
