# Supplementary material for: Dynamics of Adaptation in Spatially Heterogeneous Metapopulations
Source: PLoS One. 2013 Feb 12;8(2):e54697. doi: 10.1371/journal.pone.0054697 (PMC3570538; doi:10.1371/journal.pone.0054697)
Supplement: Appendix S3 — Hierarchical metapopulation. (PDF) [file pone.0054697.s008.pdf]

Supporting information: Dynamics of adaptation in spatially heterogeneous metapopulations

J. Papaïx [1,2,a], O. David [2,b], C. Lannou [1,c] & H. Monod [2,d]

26/12/2012

[1]INRA, UMR 1290 BIOGER, F-78850 Thiverval Grignon.

[2]INRA, UR 341 Mathématiques et Informatique Appliquées, F-78350 Jouy-en-Josas.

[a]julien.papaix@jouy.inra.fr

[b]olivier.david@jouy.inra.fr

[c]christian.lannou@grignon.inra.fr

[d]herve.monod@jouy.inra.fr

Appendix S3: hierarchical metapopulation

We consider a metapopulation composed of p_1 groups of p_2 patches, where all the patches have the same relative size $\overline{K}_j = 1/P$. Dispersal is symmetric, with three dispersal rates m_0, m_1 and m_2 defined as follows :

- $m_{j'j} = m_0$ if patches j' and j belong to two different groups,
- $m_{j'j} = m_0 + m_1$ if patches j' and j belong to the same group and $j' \neq j$,
- $m_{j'j} = m_0 + m_1 + m_2$ if patches j' and j are the same ($j' = j$).

We consider that all propagules land in one patch so that the rates satisfy $\sum_j m_{j'j} = p_1 p_2 m_0 + p_2 m_1 + m_2 = 1$ and $m_{+j} = 1/P$. Under these assumptions, the matrix A_{env} is equal to the symmetric dispersal matrix M defined in the main text (Section 2.2.1), so that

$$A_{\text{env}} = m_0\, J_P + m_1\, I_{p_1} \otimes J_{p_2} + m_2\, I_P,$$

where J_P is the $P \times P$ matrix of ones, I_P is the identity matrix of order P, $I_{p_1} \otimes J_{p_2}$ denotes the block-diagonal matrix with diagonal matrices J_{p_2} and the symbol $\otimes$ denotes the Kronecker product. The matrix A_{env} can be decomposed into

$$A_{\text{env}} = S_1 + (m_2 + p_2 m_1) S_2 + m_2 S_3, \tag{1}$$

where $S_1 = \frac{1}{p_1} J_{p_1} \otimes \frac{1}{p_2} J_{p_2}$, $S_2 = (I_{p_1} - \frac{1}{p_1} J_{p_1}) \otimes \frac{1}{p_2} J_{p_2}$, $S_3 = I_{p_1} \otimes (I_{p_2} - \frac{1}{p_2} J_{p_2})$ are the orthogonal projection matrices on three mutually orthogonal subspaces of $\mathbb{R}^P$ of dimensions $1, p_1 - 1, p_1(p_2 - 1)$ respectively. This decomposition shows that the eigenvalues of A_{env} are 1 with multiplicity one, $m_2 + p_2 m_1$ with multiplicity $p_1 - 1$ and m_2 with multiplicity $p_1(p_2 - 1)$. The dominant eigenvalue $\lambda^{(1)}_{\text{env}}$ equals one, as expected, and the normalised dominant eigenvectors respecting the scaling constraints are $r^{(1)}_{\text{env}} = 1_P/P$ and $l^{(1)}_{\text{env}} = 1_P$, where 1_P is the vector of ones of length P.

These results imply that $x^\star = (1/P) \sum_{j=1}^{P} \beta_{h(j)}$. Decomposition (1) also implies that

$$\sum_{j, j \neq 1} \frac{\lambda^{(j)}_{\text{env}}}{1 - \lambda^{(j)}_{\text{env}}} r^{(j)}_{\text{env}} l^{(j)\prime}_{\text{env}} = \rho S_2 + \xi S_3$$

and so

$$D^{(2)}_{\text{loc}}(x^\star) = \frac{1}{\sigma^2} \Big(w' \big(I_P + 2\rho S_2 + 2\xi S_3 \big) w - 1 \Big),$$

where $\rho = \dfrac{m_2 + p_2 m_1}{p_1 p_2 m_0}$, $\xi = \dfrac{m_2}{p_2 m_1 + p_1 p_2 m_0}$ and w is the vector of length P with jth coordinate equal to $(\beta_{h(j)} - x^\star)/(\sqrt{P}\sigma)$. For $g = 1, \cdots, p_1$ and $l = 1, \cdots, p_2$, let $w_{(gl)}$ denote the coordinate of w associated with patch l of group g and let $w_{(g\bullet)} = (1/p_2) \sum_l w_{(gl)}$. The vector w is centred thus $\sum_{g,l} w_{(gl)} = 0$. Then we have

$$\begin{aligned}
w' S_1 w &= 0, \\
w' S_2 w &= p_2 \sum_g w^2_{(g\bullet)} \\
&= \sum_k \sum_{k'} \text{Cov}(\pi_k, \pi_{k'}) \frac{(x^\star - \beta_k)(x^\star - \beta_{k'})}{\sigma^2}, \\
w'(S_2 + S_3) w = w' I_P w &= \sum_{g,l} w^2_{(gl)} \\
&= \sum_{k=1}^{H} \pi_k \frac{(x^\star - \beta_k)^2}{\sigma^2}.
\end{aligned}$$

We note that $w'S_2w \geq 0$ since $w'S_2w$ is a quadratic form of a positive semidefinite matrix. After setting $\nu = \rho - \xi$, we get

$$D^{(2)}_{\mathrm{loc}}(x^\star) = \frac{1}{\sigma^2}\left((1+2\xi)\sum_{k=1}^{H}\pi_k\frac{(x^\star-\beta_k)^2}{\sigma^2} + 2\nu\sum_{k=1}^{H}\sum_{k'=1}^{H}\mathrm{Cov}(\pi_k,\pi_{k'})\frac{(x^\star-\beta_k)(x^\star-\beta_{k'})}{\sigma^2} - 1\right).$$

It follows that $x^\star$ is an evolutionarily stable strategy if:

$$(1+2\xi)\sum_{k=1}^{H}\pi_k\frac{(x^\star-\beta_k)^2}{\sigma^2} + 2\nu\sum_{k=1}^{H}\sum_{k'=1}^{H}\mathrm{Cov}(\pi_k,\pi_{k'})\frac{(x^\star-\beta_k)(x^\star-\beta_{k'})}{\sigma^2} < 1.$$

When dispersal is homogeneous ($m_1 = m_2 = 0$), the condition for evolutionary stability becomes:

$$\sum_{k=1}^{H}\pi_k\frac{(x^\star-\beta_k)^2}{\sigma^2} < 1.$$

When dispersal is hindered so that a propagule is more likely to stay in its patch than to land on another patch ($m_2 \neq 0$) and when there is no group structure ($m_1 = 0$), the condition for evolutionary stability becomes:

$$(1+2\xi)\sum_{k=1}^{H}\pi_k\frac{(x^\star-\beta_k)^2}{\sigma^2} < 1.$$
