# Supplementary material for: Dynamics of Adaptation in Spatially Heterogeneous Metapopulations
Source: PLoS One. 2013 Feb 12;8(2):e54697. doi: 10.1371/journal.pone.0054697 (PMC3570538; doi:10.1371/journal.pone.0054697)
Supplement: Appendix S4 — Definition of measures on simulated evolution. (PDF) [file pone.0054697.s009.pdf]

Supporting information: Dynamics of adaptation in spatially heterogeneous metapopulations

J. Papaïx [1,2,a], O. David [2,b], C. Lannou [1,c] & H. Monod [2,d]

26/12/2012

[1]INRA, UMR 1290 BIOGER, F-78850 Thiverval Grignon.

[2]INRA, UR 341 Mathématiques et Informatique Appliquées, F-78350 Jouy-en-Josas.

[a]julien.papaix@jouy.inra.fr

[b]olivier.david@jouy.inra.fr

[c]christian.lannou@grignon.inra.fr

[d]herve.monod@jouy.inra.fr

Appendix S4: definition of measures on simulated evolution

At each time step the metapopulation composition was summarised by the means and standard deviation of a two-component Gaussian mixture that was fitted by the EM algorithm (Meng and Rubin, 1993) using the function *normalmixEM* of the R package *mixtools* (Young et al., 2010, Version 0.4.4.). The time to reach the singular strategy (T_{branch}) was defined as the first time step when two distinct populations were identified. The estimated singular strategy ($\hat{x}^{\star}$) was then defined as the mean metapopulation phenotype at $t = T_{\text{branch}} - 1$. Trait values of specialists (ESS) were estimated by the means of the two Gaussian mixture components at the end of evolution. The time to reach the specialists (T_{ESS}) was defined as the first time step when the means of the two Gaussian mixture components were contained in the $\pm 5\%$ interval around the trait values of the specialist strategies. Figure S1 of this appendix illustrates these measures on an example.

Finally, in patch j, the mean phenotype was calculated as a weighted average of the existing

phenotypes, the weights being the proportions of each phenotype in the local population:

$$\bar{x}_j(t) = \sum_{i=1}^{I} \frac{n_{ij}(t)}{K_j} x_i.$$

Then we defined the level of local adaptation in patch j as the mismatch between the mean phenotype ($\bar{x}_j(t)$) and the optimal phenotype ($\beta_{h(j)}$): $|\bar{x}_j(t) - \beta_{h(j)}|$.

References

X.L. Meng and D.B. Rubin. Maximum likelihood estimation via the ECM algorithm: a general framework. *Biometrika*, 80:267–278, 1993.

D. Young, T. Benaglia, D. Chauveau, R. Elmore, T. Hettmansperger, D. Hunter, H. Thomas, and F. Xuan. Tools for analyzing finite mixture models. Technical report, R CRAN, 2010, Version 0.4.4.
