# Supplementary material for: Dynamics of Adaptation in Spatially Heterogeneous Metapopulations
Source: PLoS One. 2013 Feb 12;8(2):e54697. doi: 10.1371/journal.pone.0054697 (PMC3570538; doi:10.1371/journal.pone.0054697)
Supplement: Appendix S5 — Comparing theoretical predictions and simulation results. (PDF) [file pone.0054697.s010.pdf]

Supporting information: Dynamics of adaptation in spatially heterogeneous metapopulations

J. Papaïx [1,2,a], O. David [2,b], C. Lannou [1,c] & H. Monod [2,d]

26/12/2012

[1]INRA, UMR 1290 BIOGER, F-78850 Thiverval Grignon.

[2]INRA, UR 341 Mathématiques et Informatique Appliquées, F-78350 Jouy-en-Josas.

[a]julien.papaix@jouy.inra.fr

[b]olivier.david@jouy.inra.fr

[c]christian.lannou@grignon.inra.fr

[d]herve.monod@jouy.inra.fr

Appendix S5: comparing theoretical predictions and simulation results

Theoretical predictions and simulation results were compared by fixing the parameters at $m_s = 75\%$, $\delta/\sigma = 0.9$ ($\delta = -\beta_1 = \beta_2 = 0.9$ and $\sigma = 1$) and $\pi = 0.32$ in order to observe both stable and unstable conditions for the generalist when habitat aggregation varied.

Although the monomorphic assumption of the analytical results was not imposed in the simulations, simulation results were highly consistent with analytical predictions. The estimated singular strategy $\hat{x}^\star$ was in line with the theoretical singular strategy $x^\star$. Indeed the mean of $\hat{x}^\star$ was close to $x^\star$ whatever the value of the aggregation of habitat 2 (Figure S2a of this appendix). It was lower than $x^\star$ for AI values between 0.6 and 0.8, because our T_{branch} criterion detected branching before population average was completely stabilised. From thorough visual inspection of the dynamics as given in Figure S1, it appears that the increasing variance observed on the estimated singular

strategy mostly reflects the difficulty to estimate $x^\star$ on simulations when a branching point was observed.

When the branching criterion was greater than 1, a branching point was observed in 90.2% of the simulations (Figure S2b of this appendix). Reciprocally, no branching was observed when the branching criterion was lower than 1. Differences between predictions based on the branching criterion and observed branchings in simulations were more important when AI values were between 0.5 and 0.6. At these values, the observed proportion of branching events was always lower than predicted. This could be explained by the stochasticity of mutations and their higher number (Section 1.3.3. of the main text) that could delay specialisation and favour the generalist for situations where the branching criterion was close to 1. In addition, the variance of phenotypic morph populations was greater and the phenotypic morph populations were less differentiated for a branching criterion close to 1 (Section 2.3 of the main text and Figure S4), which could render the detection of branching events more difficult. Further investigations are required to try and disentangle these effects.
